# An Update on Eight “New” Antibiotics against Multidrug-Resistant Gram-Negative Bacteria

**DOI:** 10.3390/jcm10051068

**Published:** 2021-03-04

**Authors:** Erlangga Yusuf, Hannelore I. Bax, Nelianne J. Verkaik, Mireille van Westreenen

**Affiliations:** Department of Medical Microbiology and Infectious Diseases, Erasmus University Medical Center, 3015 GD Rotterdam, The Netherlands; h.i.bax@erasmusmc.nl (H.I.B.); n.j.verkaik@erasmusmc.nl (N.J.V.); m.vanwestreenen@erasmusmc.nl (M.v.W.)

**Keywords:** gram-negative bacteria, new antibiotics, ESBL, CPE

## Abstract

Infections in the ICU are often caused by Gram-negative bacteria. When these microorganisms are resistant to third-generation cephalosporines (due to extended-spectrum (ESBL) or AmpC beta-lactamases) or to carbapenems (for example carbapenem producing Enterobacteriales (CPE)), the treatment options become limited. In the last six years, fortunately, there have been new antibiotics approved by the U.S. Food and Drug Administration (FDA) with predominant activities against Gram-negative bacteria. We aimed to review these antibiotics: plazomicin, eravacycline, temocillin, cefiderocol, ceftazidime/avibactam, ceftolozane/tazobactam, meropenem/vaborbactam, and imipenem/relebactam. Temocillin is an antibiotic that was only approved in Belgium and the UK several decades ago. We reviewed the in vitro activities of these new antibiotics, especially against ESBL and CPE microorganisms, potential side effects, and clinical studies in complicated urinary tract infections (cUTI), intra-abdominal infections (cIAI), and hospital-acquired pneumonia/ventilator-associatedpneumonia (HAP/VAP). All of these new antibiotics are active against ESBL, and almost all of them are active against CPE caused by KPC beta-lactamase, but only some of them are active against CPE due to MBL or OXA beta-lactamases. At present, all of these new antibiotics are approved by the U.S. Food and Drug Administration for cUTI (except eravacycline) and most of them for cIAI (eravacycline, ceftazidime/avibactam, ceftolozane/tazobactam, and imipenem/relebactam) and for HAP or VAP (cefiderocol, ceftazidime/avibactam, ceftolozane/tazobactam, and imipenem/relebactam).

## 1. Introduction

Two-thirds of the infections in intensive care units (ICUs) are caused by Gram-negative bacteria [1], and resistance of these bacteria to certain antibiotics poses a significant problem. Several multidrug resistant, Gram-negative microorganisms such as carbapenem-resistant *Acinetobacter baumannii*, *Pseudomonas* *aeruginosa,* and Enterobacterales are duly included in the recent WHO list of high-priority pathogens [2].

Beta-lactam antibiotics—penicillins, cephalosporins, carbapenems, and to a lesser extent monobactams—are often the antibiotics of choice in the ICU, but they can be inactivated by beta-lactamase enzymes (the most common and important mechanism of resistance in Gram-negative bacteria). These beta-lactamases can be classified into four molecular classes: A (for example, KPCs that confer resistance to cephalosporins and to all carbapenems, and extended-spectrum beta-lactamases (ESBLs) that confer resistance to cephalosporins); B (metallo-beta-lactamases (MBLs), such as NDM, VIM, and IMP, which can lead to resistance to all carbapenems except monobactam); C (for example, AmpC (mostly chromosomal but can also be plasmidal), which confer resistance to cephalosporins); and D (for example, OXAs that confer resistance mostly to carbapenems) [3].

Among the beta-lactam antibiotics, carbapenems have the widest spectrum of activity and are often the antibiotic class of choice when Gram-negative bacteria produce ESBL or AmpC beta-lactamases. Harbouring genes encoding ESBL and AmpC beta-lactamases confer resistance to all members of penicillin, cephalosporines up to “third” generation, and aztreonam (a monobactam antibiotic). Resistance to carbapenems poses thus a big problem. Carbapenem nonsusceptibility can be due to carbapenemase production or other mechanisms. The distinction is important from the infection-prevention point of view because carbapenemase production refers to resistance caused by beta-lactamase production encoded by transmissible genes, and not due to, for example, efflux pumps. These transmissible genes can be possessed by Enterobacterales (denoted as carbapenem-producing Enterobacterales, CPE), or by nonfermenting Gram-negative bacteria such as *Acinetobacter* spp. and *Pseudomonas aeruginosa* [4]. There is a geographic diversity in the distribution of these CPE genes. OXA-48 is most common in Europe; KPC in the US, South America and China; NDM in South Asia; and VIM in Australia [5,6,7]. To treat carbapenem-nonsusceptible microorganisms, colistin is often used. However, these microorganisms can also develop resistance to colistin; for example, by means of the *mcr-1* gene [8].

In the last five years, there have been several new antibiotics with predominant activity against Gram-negative bacteria approved by the U.S. Food and Drug Administration (FDA) and the European Medical Agency (EMA), i.e., plazomicin, eravacycline, cefiderocol; and antibiotics that combine beta-lactam with beta-lactamase inhibitors, i.e., ceftazidime/avibactam, ceftolozane/tazobactam, meropenem/vaborbactam, and imipenem-cilastatin/relebactam. Next to these new antibiotics, temocillin, a beta-lactam antibiotic effective against Gram-negative bacteria that is only approved in Belgium and the United Kingdom, is also worthy of mention.

This narrative review aims to analyze the spectrum (especially against ESBL and CPE microorganisms), potential side effects, and clinical studies of these antibiotics, with a special focus on infections in critically ill patients with complicated urinary tract infections (cUTI), intra-abdominal infections (cIAI), or hospital-acquired pneumonia/ventilator-associated pneumonia (HAP/VAP).

## 2. Methods

Newly approved (between 2015 and 2020) antibiotics with predominant activity against Gram-negative pathogens were identified from the FDA and EMA website. A subsequent search was performed in MEDLINE up to 1 December 2020, to search for information regarding surveillance studies on the activity of these antibiotics against Enterobacterales, *Pseudomonas,* and *Acinetobacter* isolates, including those that are ESBL producers or carbapenem-nonsusceptible. The results of this search are presented in Tables S1 and S2. MEDLINE and clinical trial register (clinicaltrials.gov (accessed on 1 March 2021)) searches were performed to find clinical studies on these antibiotics. We included studies performed in adult patients only. The results of the included clinical trials are presented in Table 1 (cUTI), Table 2 (cIAI), Table 3 (HAP/VAP), and of the included observational studies in Table 4.

## 3. Results

### 3.1. Plazomicin

Plazomicin is a synthetic aminoglycoside [9], and like other aminoglycosides, it inhibits bacterial protein synthesis and has dose-dependent bactericidal activity in vitro [10]. The FDA approved plazomicin (brand name Zemdri) in 2018 for use in cUTI and pyelonephritis at a dose of 15 mg/kg IV, QD. The plazomicin FDA package insert includes nephrotoxicity and ototoxicity as possible side effects. However, a pooled analysis of three studies on plazomicin showed that nephrotoxicity in patients receiving plazomicin was comparable to nephrotoxicity in comparator drugs (4.8% vs. 4.1%) [11].

In vitro, plazomicin is active against > 95% of Enterobacterales isolates, but only in 30 to 40% of *Acinetobacter* spp. or *P. aeruginosa* isolates originated from North America and Europe (i.e., susceptible at FDA susceptibility breakpoint ≤ 2 mg/L) [12,13]. It is active against ESBL isolates, and against 84.6% [12] to 97.6% [14] of carbapenem-resistant isolates. However, it should be noted that in [14], only eight isolates with OXA-48 beta-lactamase were included. In [12], 87% of 54 Enterobacterales with OXA-48 beta-lactamases were susceptible to plazomicin. Considering genes coding for carbapenemases, plazomicin is associated with good activity against KPC gene-harbouring isolates (i.e., 92.9% susceptible at ≤ 2 mg/L), less activity against OXA-48 (87%), and limited activity (40.5%) against MBL-harbouring isolates [15]. The presence of aminoglycoside-modifying enzymes does not inactivate plazomicin [16], and it can still be used in 52.2% of isolates that are resistant to three members of the aminoglycosides [15]. In comparison to other aminoglycosides, plazomicin is more active against colistin-resistant Enterobacterales (including those harbouring *mcr-1* genes), with 89.5% of isolates reported as susceptible (vs. 16.8%, 47.4%, and 63.2% susceptibility to amikacin, gentamicin, and tobramycin, respectively) [17].

Two clinical studies have been conducted in patients with complicated urinary tract infection (cUTI) (Table 1) comparing plazomicin 15 mg/kg IV, QD with meropenem 1 g IV, TID [18] and levofloxacin 750 mg IV, QD for up to 10 days [19]. Both studies showed noninferiority of plazomicin. In these studies, only a limited number of patients were included with carbapenem resistant Enterobacterales (CRE) or *P. aeruginosa*. Another randomized control trial (RCT) that was stopped prematurely due to slow enrollment of patients with bloodstream infection and bacterial pneumonia due to CRE showed that death from any cause at 28 days or clinically significant disease-related complications occurred in 24% of patients who received plazomicin 15 mg/kg IV, QD, versus 50% of patients receiving colistin (5 mg colistin base/kg QD) in combination with meropenem or tigecycline for 7 to 14 days [20].

### 3.2. Eravacycline

Eravacycline is a fluorocycline of the tetracycline class. Like other tetracyclines, it inhibits bacterial protein synthesis. The FDA approved eravacycline (brand name Xerava) in 2018 for the treatment of cIAI at a dose of 1 mg/kg IV, BD for a total duration of 4 to 14 days. It may cause the same adverse events as other tetracyclines, such as hypersensitivity reactions and permanent tooth discoloration [29]. Further, the most common adverse events are infusion-site reactions (7.7%), nausea (6.5%), vomiting (3.7%), and diarrhea (2.3%) [29].

Surveillance studies show that eravacycline is active against *E. coli* (MIC50/90: 0.12/0.5), including ESBL *E. coli* (0.25/0.5); and *K. pneumoniae* (0.25/0.5), including ESBL *K. pneumoniae* (0.06/0.5) [30]. It is active against *Acinetobacter* spp. (0.06/0.5), but has limited activity against *P. aeruginosa* (8/16) [30]. Interestingly, its minimum inhibitory concentration (MIC) correlate well with tigecycline (another tetracycline antibiotic), but it is two- to fourfold more potent [30,31]. Eravacycline is active against CRE isolates [32]. A small study shows that it is also active against colistin-resistant *E. coli* harbouring *mcr-1* [33].

In two separate RCTs, eravacycline 1.5 mg/kg IV, BD was shown to be noninferior to meropenem 1 g IV, TD [34] or ertapenem 1 g IV, QD [35] (Table 2) in treating cIAI patients (>80% of the patients also underwent surgery). The majority of pathogens in these studies were Gram-negative (>70%), including ESBL- and CPE-producing isolates, but only a limited number of infections (<10%) were due to *P. aeruginosa*. In patients with ESBL-producing Enterobacterales, clinical cure rates were 87.5% (14/16) and 84.6% (11/13) in the eravacycline and meropenem groups, respectively [34]. However, the cure rate in those patients was also > 90%, emphasizing the importance of surgery. Eravacycline has been also investigated in cUTI. Two RCTs on the clinicaltrials.gov (accessed on 1 March 2021) trials register compared eravacycline with ertapenem (clinical trial register number NCT03032510) and levofloxacin (NCT01978938) in cUTI (Table 1). Both studies showed lower cure rates (84.8% vs. 94.8%) and (60.4% vs. 66.9%) than the comparators, respectively. In both studies, the eravacycline dose was 1.5 mg/kg IV, QD. No information regarding pathogens causing UTI could be found from these two studies, and no explanation can be given regarding why the latter study showed lower clinical response in both the treatment arm and control group. A small observational study including 35 ICU patients mainly with cIAI and pneumonia with a median Acute Physiology And Chronic Health Evaluation (APACHE) score of 16 showed absence of 30-day recurrence of 91%, but only 57% resolution of signs and symptoms at 30-day follow-up [36].

### 3.3. Temocillin

Temocillin is a derivative of ticarcillin, a penicillin antibiotic targeting PBP3 primarily, that was developed and marketed in the UK in the 1980s, but was immediately abandoned because of its lack of activity against Gram-positive microorganisms, nonfermenters (including *A. baumannii* and *P. aeruginosa*), and anaerobes [42]. However, there has been a renewed interest in this antibiotic agent in the last decade as a carbapenem-sparing option, given the increasing incidence of infections with Enterobacterales resistant to third-generation cephalosporins.

Temocillin is unaffected by ESBL and AmpC, but is affected by OXA-48 and MBL [42,43,44,45]. There is no breakpoint for temocillin available from the two largest bodies that set up breakpoints for antibiotics, the European Committee for Antimicrobial Susceptibility Testing (EUCAST) or the Clinical and Laboratory Standards Institute (CLSI). The British Society for Antimicrobial Chemotherapy (BSAC) publishes two breakpoints, one for infections of the urinary tract (≤32 mg/L), and the other for systemic infection (≤8 mg/L) [45]. When the BSAC breakpoint for urine is used, temocillin shows activity against KPC-producing bacteria [45].

It has been suggested that temocillin may be used for the treatment of UTI, but so far, no results of RCTs are available. One observational study in UTI and BSI caused by ESBL or derepressed AmpC beta-lactamase-producing Enterobacterales showed clinical cure of 86% and microbiological cure of 84% [43]. In the clinical trial register, there are two ongoing studies (NCT03543436 and registry number NCT04478721) identified that compare temocillin to carbapenems for the treatment of treat cUTI due to Gram-negative bacteria resistant to third-generation cephalosporins.

### 3.4. Cefiderocol

Cefiderocol (brand name Fetroja) is a novel catechol-substituted siderophore [46]. It was approved by the FDA in 2019 at a dose of 2 g IV, TD for the treatment of cUTI, and this indication was extended to HAP/VAP in 2020. The most frequently occurring adverse reactions of cefiderocol according to the FDA package insert were diarrhea, infusion-site reactions, constipation, rash, candidiasis, cough, elevations in liver tests, headache, hypokalaemia, nausea, hypomagnesemia, and atrial fibrillation. Due to a cause that has not been established yet, cefiderocol has an FDA label warning for higher all-cause mortality versus other antibiotics in critically ill patients with multidrug-resistant Gram-negative bacterial infections, as shown in the study of Bassetti and coworkers that reported a mortality rate of 34% for cefiderocol vs. 18% in the best-available therapy group [47].

Cefiderocol is active against > 90% of Enterobacterales isolates, and had MIC50/90 of 2/8 mg/L for Enterobacterales with ESBL and AmpC [48]. It is also active against > 90% of *Acinetobacter* spp. and *P. aeruginosa* isolates [49], including those which are carbapenem-resistant [50].

A clinical trial comparing cefiderocol to imipenem-cilastatin in patients with cUTI for a median treatment duration of 9 days showed significantly higher clinical and microbiological response than in the group on imipenem (73 vs. 55%) (Table 1) [21]. In this study, the most common pathogens were *E. coli* and *Klebsiella* spp. (no information on their susceptibility to the third-generation cephalosporins was available), and 7% *P. aeruginosa*. A fresh from the press RCT comparing cefiderocol 2 g IV, TID in 145 patients with meropenem 2 g IV, TID in 146 nosocomial pneumonia patients showed comparable mortality at day 14, i.e., 12.4% vs. 11.6% [51]. A small observational study including 10 critically ill patients with bacteraemia and VAP caused by carbapenem-resistant (MIC ≥ 2 mg/L) *A. baumannii*, *Stenotrophomonas maltophilia,* or NDM-producing *K. pneumoniae* [52] who experienced clinical failure on previous antibiotic (including colistin) showed 70% clinical success at 30 days. Clinical success in this study was defined as survival, resolution of signs and symptoms, and no recurrent infection and no microbiological failure.

### 3.5. Beta-Lactam/Beta-Lactamase Inhibitor

The following antibiotics are a combination of a cephalosporin or a carbapenem antibiotic with a beta-lactamase inhibitor (BLI). By inhibiting beta-lactamases, the partner beta-lactam antibiotic is allowed to reach its target, the penicillin-binding proteins (PBPs). The BLIs that are partnered with new beta-lactam antibiotics reviewed here are tazobactam (partner to ceftolozane), avibactam (to ceftazidime), vaborbactam (to meropenem), and relebactam (to imipenem-cilastatin). Tazobactam was invented in the early 1990s, and it inhibits ESBL, but not AmpC, KPC, MBL, or OXA-48 [53]. Other BLIs were invented after 2010. Avibactam protects partner antibiotics against ESBL, AmpC, KPC, and OXA-48, but not MBL (Table 5) [53]. Relebactam is structurally related to avibactam and has the same spectrum as avibactam [54]. Vaborbactam (boronate BLI) inhibits ESBL, AmpC, and KPC, but it does not inhibit OXAs or MBLs. The addition of BLI leads to a more active antibiotic. For example, ceftazidime/avibactam is more active than ceftazidime alone against *E. coli* (MIC50/90: 0.12/0.25, vs. 0.25/32), and against *P. aeruginosa* (MIC50/90: 2/8, vs. 2/32) [55]. Another example is the addition of relebactam, which improves the activity of imipenem against most Enterobacterales (reducing the MIC by 2- to 128-fold) and *P. aeruginosa* (reducing the MIC by 8-fold) [56]. The addition of vaborbactam reduces MIC 2- to > 1024-fold and improves the activity of meropenem against most species of Enterobacterales [56].

### 3.6. Ceftazidime/Avibactam

The FDA approved ceftazidime avibactam (brand name Avycaz) in 2015 for treating cIAI (in combination with metronidazole) and cUTI, at a dose of 2.5 g IV, TD, and extended to HAP/VAP in 2018. According to the FDA package insert, ceftazidime/avibactam had comparable possible adverse events to those of ceftazidime alone; the most commonly reported adverse reactions (in  ≥5% of patients) were nausea and diarrhea, and positive direct Coombs test.

Ceftazidime/avibactam shows activity against Gram-negative bacteria producing ESBL, carbapenem nonsusceptible Enterobacterales [57], and *P. aeruginosa* [58] as long as they are not due to the presence of MBL genes. Data on the susceptibility of *Acinetobacter* to ceftazidime/avibactam is limited.

Among the “new” antibiotics in this manuscript, the most clinical data are available for ceftazidime/avibactam. In treating cUTI due to ceftazidime-resistant Enterobacterales and *Pseudomonas aeruginosa*, it was shown to be noninferior against the best-available therapy (mostly carbapenem) (Table 1) [22]. This study also included < 10% patients with cIAI. In treating cIAI (Table 2), ceftazidime/avibactam 2 g/0.5 g IV, TD, together with metronidazole showed slightly lower clinical cure at 28 to 35 days after randomization in comparison with meropenem 1 g, IV, TD: 81.6% vs. 85.1%, but the difference fell under the noninferiority margin [38]. In this study, 13.5% had a ceftazidime-resistant aerobic *E. coli* or *K. pneumoniae*. Ceftazidime/avibactam 2 g/0.5 g IV, TD has also been shown to be noninferior to meropenem 1 g IV, TD, in treating nosocomial pneumonia, including VAP (Table 3) [59], where clinical cure at 21 to 25 days after randomization was 68.8% vs. 73.0%. In this study, 28% of the responsible pathogens were nonsusceptible to ceftazidime. In treating infections due to carbapenem-resistant Enterobacterales, a meta-analysis that included RCTs and observational studies of various infection showed higher clinical cure in patients treated with ceftazidime/avibactam (relative risk (RR) = 1.61, 95% CI 1.13–2.29) and reduced mortality (RR = 0.29, 95% CI 0.13–0.63) than comparators [60]. Stone et.al. [61] showed in a post hoc study of already-published clinical trials that ceftazidime/avibactam in comparison with comparator had comparable response for cUTI caused by multidrug-resistant (MDR) Enterobacterales (76.5% (*n* = 285 patients) vs. 60.6% (*n* = 287)) and MDR *P. aeruginosa* (67.9% (*n* = 28) vs. 71.4% (*n* = 14)). The same held true for cIAI due to MDR Enterobacterales (81.8% (*n* = 176) vs. 87.5% (*n* = 200)) and MDR *P. aeruginosa* (100% (*n* = 5) vs. 100% (*n* = 7)). The number of VAP for MDR Enterobacterales was 77.1% (*n* = 48) vs. 70.9% (*n* = 55) and for MDR *P. aeruginosa* was 34.8% (*n* = 23) vs. 22.2% (*n* = 18).

### 3.7. Ceftolozane/Tazobactam

Ceftolozane/tazobactam is an antipseudomonal cephalosporin partnered with BLI tazobactam. This antibiotic combination was approved by the FDA (brand name Zerbaxa) in 2014 for cUTI and cIAI indications at a dose of 1.5 g IV, TD. The indication was extended to HAP/VAP in 2019. Like ceftazidime/avibactam, possible adverse events that are most commonly reported (in  ≥5% of patients) were nausea and diarrhea, according to the FDA package insert.

Ceftolozane alone is already active against ESBL Enterobacterales and carbapenem-nonsusceptible *P. aeruginosa* [64]. However, it has limited activity against carbapenem-nonsusceptible *Acinetobacter* spp. [65] and Enterobacterales [66]. It shows comparable activities as other anti-Pseudomonal antibiotics in *P. aeruginosa* isolates originated from Western Europe, i.e., 94.1% (vs. 79.7% ceftazidime, 76.7% piperacillin/tazobactam, 67.4% levofloxacin, 79.0% meropenem) [67].

Two RCTs, one in cIAI (Table 2) [39], and one in nosocomial pneumonia patients (Table 3) [62], showed noninferiority of ceftolozane/tazobactam in comparison with comparator antibiotics. Lucasti and coworkers compared ceftolozane/tazobactam 1 g/0.5 g IV, TD (together with metronidazole 500 mg IV, TD in treating cIAI that included ICU patients [39]. Kollef and coworkers compared ceftolozane/tazobactam 2 g/1 g IV, TD with meropenem 1 g IV, TD to treat nosocomial pneumonia patients (71% were ICU patients) [62], of whom 25% were due to *P. aeruginosa*, but the susceptibility pattern was not specified. Several observational studies (Table 4) have been published on ceftolozane/tazobactam in ICU patients and some included patients with MDR or XDR *P. aeruginosa*. Pogue and coworkers compared 100 patients who received ceftolozane/tazobactam with 100 patients who received polymyxin (*n* = 56) or aminoglycoside-based therapy (*n* = 44) to treat various infections (VAP, UTI, and HAP) due to MDR or XDR *P. aeruginosa* [68]. They showed higher clinical cure in patients treated with ceftolozane/tazobactam: 81% vs. 61% (OR of 2.7 (95% CI 1.4 to 5.2).

### 3.8. Meropenem/Vaborbactam

The FDA approved meropenem/vaborbactam (brand name Vabomere) to treat cUTI at a dose of 4 g (meropenem 2 g and vaborbactam 2 g) IV, TD. According to the FDA package insert, the adverse reactions occurring in ≥ 3% of patients treated with this antibiotic were headache, phlebitis/infusion-site reactions, and diarrhea.

The addition of vaborbactam improves the activity of carbapenem-nonsusceptible Enterobacterales, including those that harbor KPCs. However, as mentioned above, it shows limited activities against isolates with MBL and OXA [77]. Addition of vaborbactam does not improve the activity of meropenem against *A. baumannii* or *P. aeruginosa* [56].

In treating cUTI (Table 1), meropenem/vaborbactam 2 g/2 g IV, TD showed similar clinical success when it was compared with piperacillin/tazobactam 4 g/0.5 g IV, TD in a noninferiority study: 98.4% vs. 94.0% [26]. It is worth mentioning that in this study, 5% of the isolates were resistant to piperacillin/tazobactam. An interesting comparison was made between meropenem/vaborbactam (*n* = 26) for the median duration of 12 days and ceftazidime/avibactam (*n* = 105) for a median duration of 11 days in treating mostly UTI and IAI patients due to CRE in an observational study (Table 4) [74]. In this study, around half of the patients were admitted to the ICU (median APACHE II score of 27) and the patients had other antibiotics (15.4% in meropenem/vaborbactam group vs. 51%). The clinical success (survival at 30 days, resolution of symptoms and signs, sterilization of blood cultures within 7 days of treatment, and absence of recurrent infections within 90 days) was higher in meropenem/vaborbactam than in ceftazidime/avibactam group: 69.2% vs. 61.9% (*p* = 0.49). In a study with various type of infections including cUTI, HAP/VAP, and bacteremia due to carbapenem-nonsusceptible pathogens, meropenem/vaborbactam showed higher clinical cure at day 28 in comparison with the best-available therapy of 65.6% vs. 33.3% [27]. It should be noted, however, that the results might have been affected by a higher proportion of patients with Charlson comorbidity index > 6 in the best-available therapy group.

### 3.9. Imipenem-Cilastatin/Relebactam

The FDA approved imipenem-cilastatin/relebactam (brand name Recarbrio) in 2019 for cUTI and cIAI indications at a dose of 1.25 g (imipenem 500 mg, cilastatin 500 mg, and relebactam 250 mg), IV, QID. The indication was extended to HAP/VAP in 2020. Common side effects (in up to 10%) are nausea, diarrhea, elevated lever enzymes, increased eosinophils, and rash.

Like all beta-lactam/ beta-lactamase inhibitor (BL/ BLI) presented in the present manuscript, imipenem (+cilastatin)/relebactam, further referred to as imipenem/relebactam, shows activity against Enterobacterales (including ESBL and AmpC isolates) and *P. aeruginosa*, but not against *Acinetobacter* spp. [78]. It restores activity against *K. pneumoniae* isolates that harbour KPCs [79], and partnering relebactam with imipenem decreased the MIC values of imipenem in *P. aeruginosa* isolates fourfold [78]. Despite the theoretical notion that relebactam shows activity against OXAs, a surveillance study fails to show that imipenem/relebactam has activities against OXA-positive isolates [80].

An RCT that compared imipenem-cilastatin/relebactam (500 mg–500 mg/250 mg IV, QID), for 7 to 14 days with piperacillin/tazobactam 4 g/500 mg IV, QID, in treating VAP patients showed apparently lower all-cause mortality at day 28 (15.9% vs. 21.3%) [63]. In this study, the most common pathogens were Enterobacterales (41.1%), *P. aeruginosa* (18.9%), and *Acinetobacter* (15.7%) but the susceptibility patterns of these microorganisms were not mentioned. Another RCT that included patients with various type of infections (around 50% UTI) due to imipenem-resistant Gram-negative bacteria compared imipenem-cilastatin/relabactam (500 mg–500 mg/250 mg IV, QID), with 150 mg based colistin IV, BD, and imipenem-cilastatin (500 mg–500 mg IV, QID). Favorable clinical response rates at day 28 were 71.4% for the former and 40.0% for the latter in the microbiological modified intention to treat population [81].

## 4. Discussion

The eight antibiotics reviewed here add to the armamentarium of antibiotics against carbapenem-resistant Gram-negative microorganisms. Almost all of these new antibiotics are approved for cUTI (all except eravacycline) and most of them for cIAI (eravacycline, ceftazidime/avibactam, ceftolozane/tazobactam, and imipenem/relebactam). Ceftazidime/avibactam was the first antibiotic approved for HAP/VAP by the FDA in 2018, and over the years, ceftolozane/tazobactam (2019), imipenem/relebactam (2020), and cefiderocol (2020) were also approved by the FDA for this indication.

Local epidemiology of multidrug-resistant microorganisms plays a role in choosing these new antibiotics. In our setting, where the prevalence of Enterobacterales resistant to third-generation cephalosporins of less than 10%, and to carbapenem of less than 1% (EARS-Net Surveillance Atlas of Infectious Diseases 2019), these new antibiotics are clearly not suitable as an empirical treatment. Targeted treatment can be performed only after susceptibility tests (ASTs) and eventual molecular testing have been performed. Table 5 can assist in making a choice of antibiotics in treating multidrug-resistant microorganisms. There are enough options for treating cUTI, cIAI, or HAP/VAP due to KPC CPE (all except ceftolozane/tazobactam can be used). The options become more limited to treat HAP/VAP due to OXA-48 CPE (only cefiderocol and ceftazidime/avibactam), and even more limited to treat HAP/VAP due to MBL CPE (only cefiderocol), but the combination ceftazidime/avibactam with aztreonam (a monobactam antibiotic that is active against NDM) can perhaps be used, since aztreonam is active against MBL). For cIAI or HAP/VAP due to carbapenem-nonsusceptible *P. aeruginosa* caused by elevated efflux, derepressed AmpC, or porine loss, ceftazidime/avibactam or ceftolozane/tazobactam may be used. In case of HAP/VAP due to MDR *A. baumannii*, only cefiderocol is available, and perhaps eravacycline in cIAI.

In order to use these antibiotics appropriately, ASTs should be available in clinical microbiology laboratories. EUCAST has published a breakpoint for all new antibiotics except for plazomicin (and for temocillin). These breakpoints may be different than those from CLSI; for example, for plazomicin. The ASTs protocols may be also different than routine ASTs. ASTs for cefiderocol, for example, need an addition of iron in the media [84].

Surveillance studies show that many of these antibiotics are more active than other older antibiotics. For example, eravacycline is 2 to 4 times more potent (i.e., 2 to 4 times lower MIC) than tigecycline against Enterobacterales [30,31], and ceftazidime/avibactam is more active than ceftazidime alone against Enterobacterales [55]. However, in our opinion, this should not lead to the routine use of these antibiotics for the treatment of infections with Gram-negative microorganisms. We believe that these antibiotics should only be used when other options are exhausted. Inappropriate use of these new antibiotics may also lead to development of resistance.

More studies are needed that investigate the use of these antibiotics to treat infections that are caused by ESBL or carbapenem-nonsusceptible microorganisms. Many RCTs that lead to approval of these antibiotics as reviewed here often did not include these microorganisms. Exactly these microorganisms are an important reason why these new antibiotics were developed. It is also important to consider whether these antibiotics should be used alone or in combination with other antibiotics for treating an MDR bacterial infections. Combination therapy should also be considered to prevent resistance development, and for synergy when MICs are high and options are limited. The combination of these and older antibiotics should also be investigated to add to the possible therapeutic armamentarium. For example, the combination of ceftazidime/avibactam and aztreonam may be a therapeutic option against CPE due to NDM, as mentioned above. The possible combination of these new antibiotics and colistin is also interesting, since according to many surveillance studies (Tables S1 and S2), colistin is still susceptible to the carbapenem-nonsusceptible Enterobacterales, *Acinetobacter* spp., and *P. aeruginosa.*

Developing antibiotics active against Gram-negative microorganisms that harbor MBL remains a challenge. While several inhibitors for serine beta-lactamases (i.e., group A, C, and D) are available as reviewed above, not many MBL inhibitors are included. We can only speculate that this is perhaps caused by the structure of the MBL [85]. In vitro, MBL can be inhibited, for example, by aztreonam (a monobactam antibiotic) or boronic acid. There are several BL/BLI antibiotics with potential activity against MBL in clinical trials at this moment, such as aztreonam/avibactam (clinicaltrials.gov (accessed on 1 March 2021) identifier NCT03329092, in patients with cIAI and HAP/VAP; and cefepime/taniborbactam, NCT03840148, in patients with cUTI). Avibactam is partnered with aztreonam to prevent aztreonam from being hydrolyzed by non-MBL beta-lactamases. While this antibiotic seemed to be active against Enterobacterales with MBL genes (NDM, VIM, IMP), it was less active against *P. aeruginosa* with MBL genes [86]. The reason is perhaps the presence of other mechanisms than beta-lactamases.

In conclusion, we have reviewed eight novel antibiotics that are predominantly active against Gram-negative bacteria, and all have potential in the treatment of infections due to carbapenem-nonsusceptible microorganisms.

## Figures and Tables

**Table 1:** Clinical trials in complicated urinary tract infection (cUTI).

First Author (Ref)	Resistant Microorganisms ǂ	Dose New Antibiotic (*n* Patient)	Comparator, Dose (*n* Patient)	Definition Outcome	Timing Assessment of Outcomes	Outcomes (New Antibiotics vs. Comparator)
Plazomicin						
Wagenlehner [18]	ESBL 26.5% CRE 4.8%	15 mg/kg IV, QD (*n* = 306)	Meropenem 1 g IV, TID (*n* = 303)	Clinical cure and microbiological response	15 to 19 days after start of therapy	81.7% vs. 70.1%
Conolly [19]	Ceftazidime non-susceptible 17.6%	15 mg/kg IV, QD (*n* = 51)	Levofloxacin 750 mg IV, QD (*n* = 29)	Microbiological eradication rate	12 days after the last dose	60.8% vs. 58.6%
Eravacycline						
Clinical trial identifier NCT03032510	No information	1.5 mg/kg IV, QD + levofloxacin PO (*n* = 603).	Ertapenem 1 g IV, QD + levofloxacin PO (*n* = 602).	Clinical cure and microbiological response	14 to 17 days post randomization	84.8% vs. 94.8%
Clinical trial identifier NCT01978938	No information	1.5 mg/kg IV, QD (*n* = 455).	Levofloxacin 750 mg IV, QD (*n* = 453).	Clinical cure and microbiological response	Post-treatment visit	60.4% vs. 66.9%
Cefiderocol						
Portsmouth [21]	No information	2 g IV, TID (*n* = 252)	Imipenem-cilastatin 1 g IV, TID (*n* = 119)	Clinical cure and microbiological response	7 ± 2 days after end of antibiotic treatment	73%, vs. 55%
Ceftazidime/avibactam						
Carmeli [22] ^a^	Ceftazidime non-susceptible Enterobacterales or *P. aeruginosa* 100%	2 g/500 mg IV, TD (*n* = 165)	Best available therapy (97% carbapenems) (*n* = 168)	Clinical response	7 to 10 days after last infusion	91% vs. 91%
Wagenlehner [23]	Ceftazidime non-susceptible 19.6%	2 g/500 mg IV, TD (*n* = 393)	Doripenem 500 mg IV, TD (*n* = 417)	Clinical cure and microbiological response	21 to 25 days post-randomization	71.2% vs. 64.5%
Ceftolozane/tazobactam						
Popejoy [24]	ESBL 11.1%	1 g/500 mg IV, TD (*n* = 54)	Levofloxacine 750 mg IV, QD (*n* = 46) Meropenem 1 g, IV, TD (*n* = 26)	Clinical cure	5 to 9 days post therapy	95.8% vs. 82.6% (*p* = 0.01)
Wagenlehner [25]	ESBL 14.8%	1 g/500 mg IV, TD (*n* = 398)	Levofloxacine 750 mg IV, QD (*n* = 402)	Clinical cure and microbiological response	5 to 9 days post therapy	76.9% vs. 68.4%
Meropenem/vaborbactam						
Kaye [26]	Piperacillin/tazobactam-resistant *E. coli* and *K. pneumoniae* 15%	2 g/2 g IV, TD (*n* = 274)	Piperacillin/tazobactam 4 g/500 mg IV, TD (*n* = 276)	Clinical cure and microbiological response	End of intravenous treatment	98.4% vs. 94.0%
Wunderink [27] ^b^	Multicenter study (27 CRE 78.7%	2 g/2 g IV, TD (*n* = 32)	Best available therapy (*n* = 15) (46.7% dual therapy)	Cure rates	At day 28	65.6% vs. 33.3% (95%CI: 3.3. to 61.3)
Imipenem+ cilastatin/relebactam						
Motsch [28] ^c^	Imipenem-nonsusceptible microorganisms 100%	500 mg/250 mg IV, QD (*n* = 31)	Colistimethate Sodium + imipenem + cilastatin loading dose 300 mg colistin base activity, followed by maintenance doses up to 150 mg colistin base activity, IV, BD (*n* = 16)	Clinical and microbiological response Survival (HAP/VAP) Clinical response (cIAI)	On therapy visit (cUTI) At day 28 (HAP/VAP and cIAI)	71.4% vs.70.0% Favorable overall response against *P. aeruginosa:* 81% vs. 63%

Abbreviations: IV, intravenous; PO, by mouth; BD, twice daily; TID, three times daily; QD, once a day; ESBL, extended-spectrum beta-lactamases, CRE, carbapenem-resistant Enterobacterales; cUTI, complicated urinary tract infection; cIAI, complicated intra-abdominal infection; HAP/VAP, hospital-acquired pneumonia/ventilator-associated pneumonia. **ǂ** Only data on ESBL or CRE are mentioned; if total data not available, only data from new antibiotics are included; ^a^ also included patients with complicated intra-abdominal infection (<10%); ^b^ 34% cUTI patients, also included 10.6% patients with HAP/VAP and 46.8% bacteremia; ^c^ 51.6% cUTI, also included 35.5% patients with HAP/VAP, and 12.9% cIAI.

**Table 2:** Clinical trials on complicated intra-abdominal infection.

First Author (Ref)	Resistant Microorganisms *	Dose New Antibiotic (*n* Patient)	Comparator, Dose (*n* Patient)	Definition Outcome	Timing Assessment of Outcomes	Outcomes (New Antibiotics vs. Comparator)
Eravacycline						
Solomkin [34]	ESBL 9.3%	1 mg/kg IV, BD (*n* = 195, 95.4% underwent surgery).	Meropenem 1 g IV, TD (*n* = 205, 96.1% underwent surgery).	Clinical cure	25 to 31 days from start therapy	90.8% vs. 91.2% In ESBL group: 87.5% vs. 84.6%)
Solomkin [35]	ESBL 10.9%	1 mg/kg IV, BD (*n* = 270, 81.5% underwent surgery)	Ertapenem 1 g IV, QD. (*n* = 271, 100% received surgery)	Clinical cure	25 to 31 days from start therapy	87.0% vs. 88.8%
Ceftazidime/avibactam						
Qin [37]	Ceftazidime-nonsusceptible 19.7%	2 g/500 mg IV, TD + metronidazole 500 mg IV, TD (*n* = 214)	Meropenem 1 g IV, TD (*n* = 217)	Clinical cure	28 to 35 days post randomisation	93.8% vs. 94.0%
Mazuski [38]	Ceftazidime-nonsusceptible 13.5%	2 g/500 mg IV, TD + metronidazole 500 mg IV, TD (*n* = 529)	Meropenem 1 g IV, TD (*n* = 529)	Clinical cure	28 to 35 days post randomisation	81.6% vs. 85.1%
Ceftolozane/tazobactam						
Lucasti [39]		1 g/500 mg IV, TD + metronidazole 500 mg IV, TD (*n* = 61)	Meropenem 1 g IV, TD (*n* = 25)	Clinical cure	7 to 14 days after last doses	83.6% vs. 96.0%
Popejoy [24]	ESBL 11.1%	1 g/500 mg IV, TD + metronidazole 500 mg IV, TD (*n* = 24)	Meropenem 1 g IV, TD (*n* = 26)	Clinical cure	24 to 32 days post therapy	98.1% vs. 88.5%
Miller [40]	Carbapenem-nonsusceptible *P. aeruginosa* 10.1%	1 g/500 mg IV, TD + metronidazole 500 mg IV, TD (*n* = 26)	Meropenem 1 g IV, TD (*n* = 29)	Clinical cure	24 to 32 days from start therapy	100% vs. 93.1%
Solomkin [41]	ESBL 7.2%	1 g/500 mg IV, TD + metronidazole 500 mg IV, TD (*n* = 389)	Meropenem 1 g IV, TD (*n* = 417)	Clinical cure	24 to 32 days from start therapy	83.0% vs. 87.3% ESBL subgroup: 95.8% vs. 88.5%

* Only data on ESBL or CRE are mentioned; if total data not available, only data from new antibiotics are included. Abbreviations: IV, intravenous; PO, by mouth; BD, twice daily; TID, three times daily; QD, once a day; ESBL: extended-spectrum beta-lactamases, CRE: carbapenem-resistant Enterobacterales.

**Table 3:** Clinical studies on hospital-acquired or ventilator-associated pneumonia.

First Author (Ref)	Resistant Microorganisms *	Dose New Antibiotic (*n* Patient)	Comparator, Dose (*n* Patient)	Definition Outcome	Timing Assessment of Outcomes	Outcomes (New Antibiotics vs. Comparator)
Plazomicin						
McKinnell [20]	CRE 100%	15 mg/kg IV, QD (*n* = 18 patients) + meropenem or tigecycline	Colistin 5 mg/kg IV, QD (*n* = 21) + meropenem or tigecycline	Death from any cause or clinically significant disease-related complications occurred in	At 28 day	24% vs. 50%
Cefiderocol						
Wunderink [51]	ESBL 31% CRE 13%	2 g IV, TID + linezolid 600 mg IV, BD (*n* = 145)	Meropenem 2 g IV, TID + linezolid 600 mg IV, BD (*n* = 146)	All-cause mortality	Day 14	12.4% vs. 11.6%
Ceftazidime/avibactam						
Torres [59]	Ceftazidime non-susceptible 28%	2 g/500 mg IV, TD + (*n* = 356)	Meropenem 1 g IV, TD (*n* = 370)	Clinical cure	21 to 25 days post randomization	68.8% vs. 73.0%
Ceftolozane/tazobactam						
Kollef [62]	No information	2 g/1 g IV, TD (*n* = 362)	Meropenem 1 g IV, TD (*n* = 364)	All cause mortality	At 28 day	24.0% vs. 25.3%
Imipenem-cilastatin/relebactam						
Titov [63]	No information	500 mg/250 mg IV, QD (*n* = 268)	Piperacillin/tazobactam 4 g/500 mg IV, QD (*n* = 269)	All cause mortality	At 28 day	15.9% vs. 21.3%

* Only data on ESBL or CRE are mentioned; if total data not available, only data from new antibiotics are included. Abbreviations: IV, intravenous; PO, by mouth; BD, twice daily; TID, three times daily; QD, once a day; ESBL: extended-spectrum beta-lactamases, CRE: carbapenem-resistant Enterobacterales.

**Table 4:** Observational and post hoc studies on the new antibiotics.

First Author (Reference)	*n*	Type of Infections	Resistant Microorganisms	Dose (and of the Comparator, When Avaialable)	Outcomes
Eravacycline					
Alosaimy [36]	35	cIAI (35%), pneumonia (29%), bone and joint infection (14%), skin and soft tissue infection 9%)	CRE 22.9%	1 mg/kg IV, BD	30-day survival: 74%, absence of 30-day recurrence: 91% resolution of signs and symptoms of infection: 57%
Temocillin					
Balakrishnan [43]	92	46% UTI, 46% BSI, 8% HAP Non-ICU.	ESBL or derepressed AmpC resistance in 58%	2 g IV, TD	Clinical cure 86%, microbiological cure 84%
Cefiderocol					
Falcone [52]	10	Bacteraemia (60%) or VAP (40%)	Carbapenem-resistant *A. baumannii*, *S. maltophilia* or NDM-producing *K. pneumoniae*	2 g IV, TID	30-day clinical success: 70%, 30-day survival: 90%
Ceftazidime/avibactam					
Caston [69]	47	cIAI (38.3%), pneumoniae (29.8%).	*Klebsiella pneumoniae* KPC	2 g/500 mg IV, TD	14-day clinical response: 59.6% - 30-days crude mortality: 23.4% (*n* = 11)
Ceftolozane/tazobactam					
Pogue [68]	100 vs. 100	VAP (52%), UTI (14%), HAP (13%).	MDR or XDR *P. aeruginosa*	2 g/1 g IV, TD (62%), or 1 g/500 mg IV TD (38%) vs. polymyxin or aminoglycosides based therapy	Clinical cure: 81% vs. 61% (OR, 2.7 (95% CI 1.4 to 5.2)
Gallagher [70]	205	HAP/VAP (59%), cIAI, cUTI, SSTI, osteomyelitis (others).	MDR *P. aeruginosa* (all patients)	2 g/1 g IV, TD (47.3%), 1 g/500 mg IV TD (others)	Clinical cure: 73.7%, microbiological cure: 70.7%
Sheffield [71]	7	Deep-seated infections, such as infection of left ventricular assist device and ventriculoperitoneal shunt	MDR *P. aeruginosa* (all patients).	2 g/1 g IV, TD	All patients had positive outcomes
Bassetti [72]	153	HAP/VAP (30%), cUTI (22.2%), septic shock (27.5%)	ESBL *Enterobacterales* (all patients).	1 g/500 mg IV TD + concomitant antibiotics (35.6%)	Favorable clinical outcome 83.2%
Arakawa [73]	115	cUTI (100%)	ESBL 11.3%	1 g/500 mg IV TD	Favorable clinical outcome 96.6%, and composite favorable outcome (clinical and microbiological) was 80.7%
Meropenem/vaborbactam					
Ackley [74]	26 vs. 105	UTI (35%), IAI (35.5%)	CRE 100%, KPC positive 76.9%	Dose not specified + other antibiotics (15.4%) vs. ceftazidime/avibactam + other antibiotics (61.0%)	Clinical success at 30 days and absence of recurrent infections within 90 days: 69.2% vs. 61.9%
Alosaimy [75]	40	Pneumonia (32.5%), UTI (20%), IAI (12.5%), SSTI (12.5%).	CRE 84.6%	Dose not specified + other antibiotics (37.5%)	Clinical success at 30 days and absence of recurrent infections after last dose: 70%
Shields [76]	20	Bacteremia (40%), VAP (25%).	CRE 100%	2 g/2 g IV, QD + other antibiotics (20%)	Clinical success at 30 days following the onset of infection: 65%

Abbreviations: ESBL, extended-spectrum beta-lactamases; CRE, carbapenem-resistant Enterobacterales; MDR, multidrug resistant; XDR, extended-drug resistant; cIAI, complicated intra-abdominal infection; cUTI, complicated urinary tract infection; HAP, hospital-acquired pneumonia; SSTI, skin and soft tissue infection; VAP, ventilator-associated pneumonia.

**Table 5:** Possible applications of new antibiotics against Gram-negative bacteria based on resistant mechanisms.

	ESBL and AmpC	KPC	OXA-48	MBL	Carbapenem Nonsusceptible *A. baumanii*	Carbapenem Nonsusceptible *P. aeruginosa*
Plazomicin	++	++	++	+/− ^a^	−	−
Eravacycline	++	++	++	+ ^b^	++	−
Temocillin	++ (urine breakpoint only)	++ (urine breakpoint only)	−	−	−	−
Cefiderocol	++	++	++	++	++	++
Ceftazidime/avibactam	++	++	++	−	−	+/−
Ceftolozane/tazobactam	++	−	−	−	−	+/− ^c^
Meropenem/vaborbactam	++	++	−	−	?	?
Imipenem/relebactam	++	++	−	−	−	+/− ^d^

++: Activity (>90% of the isolates); +: activity in 70 to 90% of the isolates; +/−: activity in around the half of the; −: no activity; ?: no surveillance data available. ^a^ 42.1% susceptible isolates [12]; ^b^ 70% susceptible isolates [32]; ^c^ good activity against isolates with elevated efflux, derepressed AmpC or loss of OprD, but not when the underlying mechanism is MBL production [82]; ^d^ not for isolates with class B or D carbapenemase activity [83].

## Data Availability

This is a systematic review, all data are presented in (Supplementary) tables.

## Supplementary Materials

The following are available online at https://www.mdpi.com/2077-0383/10/5/1068/s1, Table S1: Susceptibility patterns of new antibiotics in large surveillance studies from various geographic regions, Table S2: Proportion (%) susceptible in various antimicrobial resistant isolates.

## Abbreviations

Regarding drugs and their use:

BD	twice daily
BL/BLI	beta-lactam, beta-lactam inhibitor.
IV	intravenous
PO	by mouth
QD	once a day
QID	four times a day
TID	three times daily

Regarding microorganisms:

CPE	carbapenem-producing Enterobacterales
CRE	carbapenem-resistant Enterobacterales
ESBL	extended-spectrum beta-lactamases
MBL	metallo-beta-lactamase.
MDR	multidrug resistance

Regarding infections:

cIAI	complicated intra-abdominal infections
cUTI	complicated urinary tract infections
HAP/VAP	hospital-acquired pneumonia/ventilator-associated pneumonia

Others:

APACHE score	Acute Physiology and Chronic Health Evaluation score
BLI	beta-lactamase inhibitor
MIC	minimum inhibitory concentration
RCT	randomized control trial
RR	relative risk.

## References

[1] Vincent J.-L., Rello J., Reinhart K., Marshall J.K., Silva E., Anzueto A., Martin C.D., Moreno R., Lipman J., EPIC II Group of Investigators (2009). International study of the prevalence and outcomes of infection in intensive care units. JAMA.

[2] Tacconelli E., Carrara E., Savoldi A., Harbarth S., Mendelson M., Monnet D.L., Pulcini C., Kahlmeter G., Kluytmans J., Carmeli Y. (2018). Discovery, research, and development of new antibiotics: The WHO priority list of antibiotic-resistant bacteria and tuberculosis. Lancet Infect. Dis..

[3] Bush K. (2018). Past and present perspectives on β-lactamases. Antimicrob. Agents Chemother..

[4] Walsh T.R., Toleman M.A., Poirel L., Nordmann P. (2005). Metallo-β-lactamases: The quiet before the storm?. Clin. Microbiol. Rev..

[5] Munoz-Price L.S., Poirel L.A., Bonomo R., Schwaber M.J., Daikos G.L., Cormican M., Cornaglia G., Garau J., Gniadkowski M., Hayden M.K. (2013). Clinical epidemiology of the global expansion of Klebsiella pneumoniae carbapenemases. Lancet Infect. Dis..

[6] Poirel L., Potron A., Nordmann P. (2012). OXA-48-like carbapenemases: The phantom menace. J. Antimicrob. Chemother..

[7] Glasner C., Albiger B., Buist G., Tambić A.A., Canton R., Carmeli Y. (2013). Carbapenemase-producing Enterobacte-riaceae in Europe: A survey among national experts from 39 countries, February 2013. Eurosurveillance.

[8] Wang R., Van Dorp L., Shaw L.P., Bradley P., Wang Q., Wang X., Jin L., Zhang Q., Liu Y., Rieux A. (2018). The global distribution and spread of the mobilized colistin resistance gene mcr-1. Nat. Commun..

[9] Shaeer K.M., Zmarlicka M.T., Chahine E.B., Piccicacco N., Cho J.C. (2019). Plazomicin: A next-generation aminoglycoside. Pharmacother. J. Hum. Pharmacol. Drug Ther..

[10] Eljaaly K., Alharbi A., AlShehri S., Ortwine J.K., Pogue J.M. (2019). Plazomicin: A novel aminoglycoside for the treatment of resistant gram-negative bacterial infections. Drugs.

[11] Tang H.-J., Lai C.-C. (2019). Plazomicin-associated nephrotoxicity. Clin. Infect. Dis..

[12] Castanheira M., Deshpande L.M., Woosley L.N., Serio A.W., Krause K.M., Flamm R.K. (2018). Activity of plazomicin compared with other aminoglycosides against isolates from European and adjacent countries, including Enterobacteriaceae molecularly characterized for aminoglycoside-modifying enzymes and other resistance mechanisms. J. Antimicrob. Chemother..

[13] Zhanel G.G., Adam H.J., Baxter M.R., Fuller J.A., Nichol K., Denisuik A.J., Golden A.R., Hink R., Lagacé-Wiens P.R.S., Walkty A. (2019). 42936 pathogens from Canadian hospitals: 10 years of results (2007–16) from the CANWARD surveillance study. J. Antimicrob. Chemother..

[14] Jacobs M.R., Good C.E., Patel R., Arias C.A., Kreiswirth B.N., Rojas L.J., D’Souza R., White R.C., Brinkac L.M., Nguyen K. (2020). Argonaut II study of the in vitro activity of plazomicin against carbapenemase-producing klebsiella pneumoniae. Antimicrob. Agents Chemother..

[15] Castanheira M., Sader H.S., Mendes R.E., Jones R.N. (2020). Activity of plazomicin tested against enterobacterales isolates collected from U.S. Hospitals in 2016–2017: Effect of different breakpoint criteria on susceptibility rates among aminoglycosides. Antimicrob. Agents Chemother..

[16] Cox G., Ejim L., Wright G.D., Stogios P.J., Koteva K., Bordeleau E., Evdokimova E., Sieron A.O., Savchenko A., Serio A.W. (2018). Plazomicin retains antibiotic activity against most aminoglycoside modifying enzymes. ACS Infect. Dis..

[17] Denervaud-Tendon V., Poirel L.E., Connolly L., Krause K.M., Nordmann P. (2017). Plazomicin activity against polymyxin-resistant Enterobacteriaceae, including MCR-1-producing isolates. J. Antimicrob. Chemother..

[18] Wagenlehner F.M.E., Cloutier D.J., Komirenko A.S., Cebrik D.S., Krause K.M., Keepers T.R., Connolly E., Miller L.G., Friedland I., Dwyer J.P. (2019). Once-daily plazomicin for com-plicated urinary tract infections. N. Engl. J. Med..

[19] Connolly L.E., Riddle V., Cebrik D., Armstrong E.S., Miller L.G. (2018). A multicenter, randomized, double-blind, phase 2 study of the efficacy and safety of plazomicin compared with levofloxacin in the treatment of complicated urinary tract infection and acute pyelonephritis. Antimicrob. Agents Chemother..

[20] McKinnell J.A., Dwyer J.P., Talbot G.H., Connolly L.E., Friedland I., Smith A., Jubb A.M., Serio A.W., Klause K.M., Daikos G.L. (2019). Plazomicin for infections caused by car-bapenem-resistant Enterobacteriaceae. N. Engl. J. Med..

[21] Portsmouth S., van Veenhuyzen D., Echols R., Machida M., Ferreira J.C.A., Ariyasu M., Tenke P., Nagata T.D. (2018). Cefiderocol versus imipenem-cilastatin for the treatment of complicated urinary tract infections caused by Gram-negative uropathogens: A phase 2, randomised, double-blind, non-inferiority trial. Lancet Infect. Dis..

[22] Carmeli Y., Armstrong J., Laud P.J., Newell P., Stone G., Wardman A., Gasink L.B. (2016). Ceftazidime-avibactam or best available therapy in patients with ceftazidime-resistant Enterobacteriaceae and Pseudomonas aeruginosa complicated urinary tract infections or complicated intra-abdominal infections (REPRISE): A randomised, pathogen-directed, phase 3 study. Lancet Infect. Dis..

[23] Wagenlehner F.M., Sobel J.D., Newell P., Armstrong J., Huang X., Stone G.G., Yates K., Gasink L.B. (2016). Ceftazidime-avibactam versus doripenem for the treatment of complicated urinary tract infections, including acute pyelonephritis: Recapture, a phase 3 randomized trial program. Clin. Infect. Dis..

[24] Popejoy M.W., Paterson D.L., Cloutier D., Huntington J.A., Miller B., Bliss C.A., Steenbergen J.N., Hershberger E., Umeh O., Kaye K.S. (2016). Efficacy of ceftolozane/tazobactam against urinary tract and intra-abdominal infections caused by ESBL-producing Escherichia coli and Klebsiella pneumoniae: A pooled analysis of Phase 3 clinical trials. J. Antimicrob. Chemother..

[25] Wagenlehner F.M., Umeh O., Steenbergen J., Yuan G., Darouiche R.O. (2015). Ceftolozane-tazobactam compared with levofloxacin in the treatment of complicated urinary-tract infections, including pyelonephritis: A randomised, double-blind, phase 3 trial (ASPECT-cUTI). Lancet.

[26] Kaye K.S., Bhowmick T., Metallidis S., Bleasdale S.C., Sagan O.S., Stus V., Vasquez J., Zaitsev V., Bidair M., Giamarellos-Bourboulis E.J. (2018). Effect of meropenem-vaborbactam vs piperacil-lin-Tazobactam on clinical cure or improvement and microbial eradication in complicated urinary tract infection the TANGO I randomized clinical trial. JAMA J. Am. Med. Assoc..

[27] Wunderink R.G., Giamarellos-Bourboulis E.J., Rahav G., Mathers A.J., Bassetti M., Vazquez J., Cornely O.A., Solomkin J., Bhowmick T., Kaye K.S. (2018). Effect and Safety of Meropenem–Vaborbactam versus Best-Available Therapy in Patients with Carbapenem-Resistant Enterobacteriaceae Infections: The TANGO II Randomized Clinical Trial. Infect Dis Ther..

[28] Motsch J., De Oliveira C.M., Du J., Joeng H.-K., Tipping R.W., Aggrey A., Young K., Kartsonis N.A., Butterton J.R., Paschke A. (2020). Restore-imi 1: A multicenter, randomized, double-blind trial comparing efficacy and safety of imipenem/Relebactam vs colistin plus imipenem in patients with imipenem-nonsusceptible bacterial infections. Clin. Infect. Dis..

[29] Heaney M., Mahoney M.V., Gallagher J.C. (2019). Eravacycline: The tetracyclines strike back. Ann. Pharmacother..

[30] Zhanel G.G., Baxter M.R., Adam H.J., Sutcliffe J., Karlowsky J.A. (2018). In vitro activity of eravacycline against 2213 Gram-negative and 2424 Gram-positive bacterial pathogens isolated in Canadian hospital laboratories: CANWARD surveillance study 2014–2015. Diagn. Microbiol. Infect. Dis..

[31] Livermore D.M., Mushtaq B.S., Warner A.M., Woodforda A.N. (2016). In vitro activity of eravacycline against carbapenem-Resistant enterobacteriaceae and acinetobacter baumannii. Antimicrob. Agents Chemother..

[32] Johnston B.D., Thuras P., Porter S.B., Anacker M., VonBank B., Vagnone P.S., Witwer M., Castanheira M., Johnsonet J.R. (2020). Activity of cefiderocol, ceftazidime-avibactam, and eravacycline against carbapenem-resistant escherichia coli isolates from the united states and international sites in re-lation to clonal background, resistance genes, coresistance, and region. Antimicrob. Agents Chemother..

[33] Fyfe C., LeBlanc G., Close B., Nordmann P., Dumas J., Grossman T.H. (2016). Eravacycline is active against bacterial isolates expressing the polymyxin resistance gene mcr-1. Antimicrob. Agents Chemother..

[34] Solomkin J.S., Gardovskis J., Lawrence K., Montravers P., Sway A., Evans D., Tsai L. (2018). Ignite4: Results of a phase 3, randomized, multicenter, prospective trial of eravacycline vs meropenem in the treatment of complicated intraabdominal infections. Clin. Infect. Dis..

[35] Solomkin J., Evans D., Slepavicius A., Lee P., Marsh A., Tsai L., Sutcliffe J.A., Horn A. (2017). Assessing the efficacy and safety of Eravacycline vs Ertapenem in complicated intra-abdominal infections in the investigating gram-negative infections treated with erava-cycline (IGNITE 1) trial a randomized clinical trial. JAMA Surg..

[36] Alosaimy S., Molina K.C., Claeys K.C., Andrade J., Truong J., King M.A., Pullinger M.B., Huang G., Morrisette T., Lagnf A.M. (2020). Early experience with eravacycline for complicated infections. Open For. Infect. Dis..

[37] Qin X., Tran B.G., Kim M.J., Wang L., Nguyen D.A., Chen Q., Song J., Laud P.J., Store G.G., Chow J.W. (2017). A randomised, double-blind, phase 3 study comparing the efficacy and safety of ceftazidime/avibactam plus metronidazole versus meropenem for complicated intra-abdominal infections in hospitalised adults in Asia. Int. J. Antimicrob. Agents..

[38] Mazuski J.E., Gasink L.B., Armstrong J., Broadhurst H., Stone G.G., Rank D., Llorens L., Newell P., Pachl J. (2016). Efficacy and safety of ceftazidime-avibactam plus metronidazole versus meropenem in the treatment of complicated intra-abdominal infection: Results from a randomized, controlled, double-blind, phase 3 program. Clin. Infect. Dis..

[39] Lucasti C., Hershberger E., Miller B., Yankelev S., Steenbergen J., Friedland I., Solomkin J. (2014). Multicenter, double-blind, randomized, phase II trial to assess the safety and efficacy of ceftolozane-tazobactam plus metronidazole compared with meropenem in adult patients with complicated intra-abdominal infections. Antimicrob. Agents Chemother..

[40] Miller B., Popejoy M.W., Hershberger E., Steenbergen J.N., Alverdy J. (2016). Characteristics and outcomes of complicated in-tra-abdominal infections involving Pseudomonas aeruginosa from a randomized, double-blind, phase 3 ceftolozane-tazobactam study. Antimicrob. Agents Chemother..

[41] Solomkin J., Hershberger E., Eckmann C., Miller B., Popejoy M., Friedland I., Steenbergen J., Yoon M., Collins S., Yuan G. (2015). Ceftolozane/Tazobactam plus metronidazole for complicated intra-abdominal infections in an era of multidrug resistance: Results from a randomized, double-blind, phase 3 trial (Aspect-ciai). Clin. Infect. Dis..

[42] Livermore D.M., Tulkens P.M. (2008). Temocillin revived. J. Antimicrob. Chemother..

[43] Balakrishnan I., Awad-El-Kariem F.M., Aali A., Kumari P., Mulla R., Tan B., Brudney D., Ladenheim D., Ghazy A., Khan I. (2011). Temocillin use in England: Clinical and mi-crobiological efficacies in infections caused by extended-spectrum and/or derepressed AmpC β-lactamase-producing En-terobacteriaceae. J. Antimicrob. Chemother..

[44] Adams-Haduch J.M., Paterson D.L., Doi Y., Potoski B.A., Sidjabat H.E. (2009). Activity of Temocillin against KPC-Producing Klebsiella pneumoniae and Escherichia coli. Antimicrob. Agents Chemother..

[45] Tsakris A., Koumaki V., Politi L., Balakrishnan I., Tsakris A. (2020). Activity of temocillin against KPC-producing Enterobacteriaceae clinical isolates. Int. J. Antimicrob. Agents.

[46] Zhanel G.G., Golden A.R., Lagacé-Wiens P.R.S., Walkty A.J., Noreddin A., Iii J.P.L., Karlowsky J.A., Zelenitsky S., Wiebe K., Lawrence C.K. (2019). Cefiderocol: A siderophore cephalosporin with activity against carbapenem-resistant and multidrug-resistant gram-negative bacilli. Drugs.

[47] Bassetti M., Echols R., Matsunaga Y., Ariyasu M., Doi Y., Ferrer R., Lodise T.P., Naas T., Niki Y., Paterson D.L. (2021). Efficacy and safety of cefiderocol or best available therapy for the treatment of serious infections caused by carbapenem-resistant Gram-negative bacteria (CREDIBLE-CR): A randomised, open-label, multicentre, pathogen-focused, descriptive, phase 3 trial. Lancet Infect. Dis..

[48] Jacobs M.R., Abdelhamed A.M., Kreiswirth B.N., Greco C., Fouts D.E., Bonomo R.A., Good C.E., Rhoads D.D., Hujer K.M., Hujer A.M. (2018). Argonaut-I: Activity of cefiderocol (S-649266), a siderophore cephalosporin, against gram-negative bacteria, including carbapenem-resistant nonfermenters and enterobacteriaceae with defined extended-spectrum β-lactamases and carbapenemases. Antimicrob. Agents Chemother..

[49] Kresken M., Korte-Berwanger M., Gatermann S.G., Pfeifer Y., Pfennigwerth N., Seifert H., Werner G. (2020). In vitro activity of cefiderocol against aerobic Gram-negative bacterial pathogens from Germany. Int. J. Antimicrob. Agents.

[50] Kresken M., Körber-Irrgang B., Pfeifer Y., Werner G. (2018). Activity of temocillin against CTX-M-producing Escherichia coli and Klebsiella pneumoniae from Germany. Int. J. Antimicrob. Agents.

[51] Wunderink R.G., Matsunaga Y., Ariyasu M., Clevenbergh P., Echols R., Kaye K.S., Kollef M., Menon A., Pogue J.M., Nagata T.D. (2021). Cefiderocol versus high-dose, extend-ed-infusion meropenem for the treatment of Gram-negative nosocomial pneumonia (APEKS-NP): A randomised, dou-ble-blind, phase 3, non-inferiority trial. Lancet. Infect. Dis..

[52] Falcone M., Tiseo G., Menichetti F., Nicastro M., Leonildi A., Vecchione A., Casella C., Forfori F., Malacarne P., Guarracino F. (2020). Cefiderocol as rescue therapy for acinetobacter baumannii and other carbapenem-resistant gram-negative infections in intensive care unit patients. Clin. Infect. Dis..

[53] van Duin D., Bonomo R.A. (2016). Ceftazidime/Avibactam and Ceftolozane/Tazobactam: Second-generation β-Lactam/β-lactamase inhibitor combinations. Clin. Infect. Dis..

[54] Wong D., van Duin D. (2017). Novel beta-lactamase inhibitors: Unlocking their potential in therapy. Drugs.

[55] Sader H.S., Flamm R.K., Carvalhaes C.G., Castanheira M. (2020). Comparison of ceftazidime-avibactam and ceftolozane-tazobactam in vitro activities when tested against gram-negative bacteria isolated from patients hospitalized with pneumonia in United States medical centers (2017–2018). Diagn. Microbiol. Infect. Dis..

[56] Zhanel G.G., Lawrence C.K., Adam H., Schweizer F., Zelenitsky S., Zhanel M., Lagacé-Wiens R.S., Walkty A., Denisuik A., Golden A. (2018). Imipenem–relebactam and meropenem–vaborbactam: Two novel carbapenem-β-lactamase inhibitor combinations. Drugs.

[57] Sader H.S., Carvalhaes C.G., Streit J.M., Doyle T.B., Castanheira M. (2020). Antimicrobial activity of ceftazidime-avibactam, ceftolozane-tazobactam and comparators tested against pseudomonas aeruginosa and klebsiella pneumoniae isolates from united states medical centers in 2016–2018. Microb. Drug Resist..

[58] Mirza H.C., Hortaç E., Koçak A.A., Demirkaya M.H., Yayla B., Güçlü A.Ü., Başustaoğlu A. (2020). In vitro activity of ceftolozane–tazobactam and ceftazidime–avibactam against clinical isolates of meropenem-non-susceptible Pseudomonas aeruginosa: A two-centre study. J. Glob. Antimicrob. Resist..

[59] Torres A., Zhong N., Pachl J., Timsit J.-F., Kollef M., Chen Z., Song J., Taylor D., Laud P.J., Stone G.G. (2018). Ceftazidime-avibactam versus meropenem in nosocomial pneumonia, including ventilator-associated pneumonia (REPROVE): A randomised, double-blind, phase 3 non-inferiority trial. Lancet Infect. Dis..

[60] Zhong H., Zhao X.-Y., Zhang Z.-L., Gu Z.-C., Zhang C., Gao Y., Cui M. (2018). Evaluation of the efficacy and safety of ceftazidime/avibactam in the treatment of Gram-negative bacterial infections: A systematic review and meta-analysis. Int. J. Antimicrob. Agents.

[61] Stone G.G., Newell P., Gasink L.B., Broadhurst H., Wardman A., Yates K., Chen Z., Song J., Chow J.W. (2018). Clinical activity of ceftazidime/avibactam against MDR Enterobacteriaceae and Pseudomonas aeruginosa: Pooled data from the ceftazidime/avibactam Phase III clinical trial programme. J. Antimicrob. Chemother..

[62] Kollef M.H., Nováček M., Kivistik Ü., Réa-Neto Á., Shime N., Martin-Loeches I., Timsit J.-F., Wunderink R.G., Burno C.J., Rhee E.G. (2019). Ceftolozane–tazobactam versus mero-penem for treatment of nosocomial pneumonia (ASPECT-NP): A randomised, controlled, double-blind, phase 3, non-inferiority trial. Lancet Infect. Dis..

[63] Titov I., Wunderink R.G., Roquilly A., Rodríguez Gonzalez D., David-Wang A., Boucher. H.W., Kaye K.S., Losada M.C., Du J., Tipping R. (2020). A randomized, double-blind, multicenter trial comparing efficacy and safety of imipenem/Cilastatin/Relebactam versus piperacil-lin/Tazobactam in adults with hospital-acquired or ventilator-associated bacterial pneumonia (Restore-imi 2 study). Clin. Infect. Dis..

[64] Shortridge D., Castanheira M., Pfaller M.A., Flamm R.K. (2017). Ceftolozane-tazobactam activity against pseudomonas aeruginosa clinical isolates from U.S. Hospitals: Report from the pacts antimicrobial surveillance program, 2012 to 2015. Antimicrob. Agents Chemother..

[65] Kuo S.-C., Liu C.-E., Lu P.-L., Chen Y.-S., Lu M.-C., Ko W.-C., Hsueh P.-R., Chuang Y.-C., Wang F.-D. (2020). Activity of ceftolozane-tazobactam against Gram-negative pathogens isolated from lower respiratory tract infections in the Asia-Pacific region: SMART 2015–2016. Int. J. Antimicrob. Agents.

[66] Karlowsky J.A., Hackel M.A., Bouchillon S.K., Sahm D.F. (2020). In vitro activity of WCK 5222 (Cefepime-zidebactam) against worldwide collected gram-negative bacilli not susceptible to carbapenems. Antimicrob. Agents Chemother..

[67] Sader H.S., Carvalhaes C.G., Duncan L.R., Flamm R.K., Shortridge D. (2020). Susceptibility trends of ceftolozane/tazobactam and comparators when tested against European Gram-negative bacterial surveillance isolates collected during 2012–18. J. Antimicrob. Chemother..

[68] Pogue J.M., Kaye K.S., Veve M.P., Patel T.S., Gerlach A.T., Davis S.L., Puzniak L.A., File T.M., Olson S., Perez F. (2020). Ceftolozane/tazobactam vs polymyxin or aminogly-coside-based regimens for the treatment of drug-resistant Pseudomonas aeruginosa. Clin. Infect. Dis..

[69] Castón J.J., Gallo M., García M., Cano A., Escribano A., Machuca I., Gracia-Aufinger I., Guzman-Puche J., Pérez-Nadales E., Recio M. (2020). Ceftazidime-avibactam in the treatment of infections caused by KPC-producing Klebsiella pneumoniae: Factors associated with clinical efficacy in a single-center cohort. Int. J. Antimicrob. Agents.

[70] Gallagher J.C., Satlin M.J., Claeys K.C., Hiles J., Vyas N.M., Bland C.M., Suh J., Biason K., McCoy D., A King M. (2018). Ceftolozane-tazobactam for the treatment of multidrug-resistant pseudomonas aeruginosa infections: A multicenter study. Open Forum Infect. Dis..

[71] Sheffield M., Nelson D., O’Neal M., Gould A.P., Bouchard J., Nicolau D., Bookstaver P.B. (2020). Use of continuous-infusion ceftolozane/tazobactam for resistant Gram-negative bacterial infections: A retrospective analysis and brief review of the literature. Int. J. Antimicrob. Agents..

[72] Bassetti M., Vena A., Giacobbe D.R., Falcone M., Tiseo G., Giannella M., Pascale R., Meschiari M., DiGaetano M., Oliva A. (2020). Ceftolozane/Tazobactam for treatment of severe ESBL-producing enterobacterales infections: A multicenter nationwide clinical experience (CEFTABUSE II Study). Open Forum Infect. Dis..

[73] Arakawa S., Kawahara K., Kawahara M., Yasuda M., Fujimoto G., Sato A., Yokokawa R., Yoshinari T., Rhee E.G., Aoyama N. (2019). The efficacy and safety of tazobac-tam/ceftolozane in Japanese patients with uncomplicated pyelonephritis and complicated urinary tract infection. J. Infect. Chemother..

[74] Ackley R., Roshdy D., Meredith J., Minor S., Anderson W.E., Capraro G.A., Polk C. (2020). Meropenem-vaborbactam versus ceftazidime-avibactam for treatment of carbapenem-resistant enterobacteriaceae infections. Antimicrob. Agents Chemother..

[75] Alosaimy S., Jorgensen S.C.J., Athans V., Saw S., Yost C.N., Davis S.L., Rybak M.J., Lagnf A.M., Melvin S., Mynatt R.P. (2020). Real-world multicenter analysis of clinical outcomes and safety of meropenem-vaborbactam in patients treated for serious gram-negative bacterial infections. Open Forum Infect. Dis..

[76] Shields R.K., McCreary E.K., Nguyen M.H., Marini R.V., Kline E.G., Jones E.C., Hao B., Chen L., Kreiswirth B.N., Doi Y. (2020). Early experience with meropenem-vaborbactam for treatment of carbapenem-resistant enterobacteriaceae infections. Clin. Infect. Dis..

[77] Castanheira M., Doyle T.B., Kantro V., Mendes R.E., Shortridge D. (2020). Meropenem-vaborbactam activity against car-bapenem-resistant enterobacterales isolates collected in U.S. Hospitals during 2016 to 2018. Antimicrob. Agents Chemother..

[78] Lapuebla A., Abdallah M., Olafisoye O., Cortes C., Urban C., Landman D., Quale J. (2015). Activity of imipenem with relebactam against gram-negative pathogens from New York city. Antimicrob. Agents Chemother..

[79] Lob S.H., Hackel M.A., Kazmierczak K.M., Hoban D.J., Young K., Motyl M.R., Karlowsky J.A., Sahm D.F. (2017). In vitro activity of imipenem-relebactam against gram-negative bacilli isolated from patients with lower respiratory tract infections in the United States in 2015–Results from the SMART global surveillance program. Diagn. Microbiol. Infect. Dis..

[80] Canver M.C., Satlin M.J., Westblade L.F., Kreiswirth B.N., Chen L., Robertson A., Fauntleroy K., La Spina M., Callan K., Jenkins S.G. (2019). Activity of imipenem-relebactam and comparator agents against genetically characterized isolates of carbapenem-resistant enterobacteriaceae. Antimicrob. Agents Chemother..

[81] Kaye K.S., Boucher H.W., Paschke A., Brown M.L., Aggrey A., Khan I., Joeng H.-K., Tipping R.W., Du J., Young K. (2020). Comparison of treatment outcomes between analysis populations in the restore-imi 1 phase 3 trial of imipenem-cilastatin-relebactam versus colistin plus imipenem-cilastatin in patients with imipenem-nonsusceptible bacterial infections. Antimicrob. Agents Chemother..

[82] Livermore D.M., Mushtaq S., Meunier D., Hopkins K.L., Hill R., Adkin R., Chaudhry A., Pike R., Staves S., Woodford N. (2017). Activity of ceftolozane/tazobactam against surveillance and “problem” Enterobacteriaceae, Pseudomonas Aeruginosa and non-fermenters from the British Isles. J. Antimicrob. Chemother..

[83] Mushtaq S., Meunier D., Vickers A., Woodford N., Livermore D.M. (2020). Activity of imipenem/relebactam against Pseudomonas aeruginosa producing ESBLs and carbapenemases. J. Antimicrob. Chemother..

[84] Simner P.J., Patel R. (2020). Cefiderocol antimicrobial susceptibility testing considerations: The Achilles’ Heel of the Trojan Horse?. J. Clin. Microbiol..

[85] Palzkill T. (2013). Metallo-β-lactamase structure and function. Ann. N. Y. Acad. Sci..

[86] Karlowsky J.A., Kazmierczak K.M., De Jonge B.L.M., Hackel M.A., Sahm D.F., Bradford P.A. (2017). In vitro activity of aztreonam-avibactam against enterobacteriaceae and pseudomonas aeruginosa isolated by clinical laboratories in 40 countries from 2012 to 2015. Antimicrob Agents Chemother..

