# Supplementary material for: An Update on Eight “New” Antibiotics against Multidrug-Resistant Gram-Negative Bacteria
Source: J Clin Med. 2021 Mar 4;10(5):1068. doi: 10.3390/jcm10051068 (PMC7962006; doi:10.3390/jcm10051068)
Supplement: Supplementary file 1 [file jcm-10-01068-s001.pdf]

Supplementary Materials

Table S1. Susceptibility patterns of new antibiotics in large surveillance studies from various geographic regions.

	**% susceptible (breakpoint used), n isolates tested, geographic origin, MIC 50/90 value (when available) in mg/L, vs. % susceptible of other antibiotics from the same study when available (reference)**		
**Antibiotics**	***Enterobacterales***	***Acinetobacter* spp.**	***Pseudomonas aeruginosa***
**Plazomicin**	97.0% (FDA, ≤2 mg/L), n=8783, USA, 0.5/1 (1)<br><br>95.8% (FDA susceptibility breakpoint ≤2 mg/L), n=4217, Europe, n.a. (2)<br><br>99.6% (FDA, ≤2 mg/L), vs.<br>>99% to amikacin, 90.5% (*E. coli)* and 96.8% (*K. pneumoniae*) to gentamicin, >99% to meropenem, >98% to colistin (3).	% n.a. (no breakpoint available), n=171, Canada, 0.5/2 (3)<br><br>40.4% (FDA susceptibility breakpoint ≤2 mg/L), n=99, Europe, n.a. (2)	% na (no breakpoint available), n=3864, Canada, 4/16 (3)<br><br>32.4% (FDA susceptibility breakpoint ≤2 mg/L), n=102, Europe, n.a. (2)
**Eravacycline**	% na (no breakpoint available), n=2067, Canada, 0.12/ 0.5 (4)	% na (no breakpoint available), n=28, Canada 0.06/ 0.5 (4)	% na, n=1647, worldwide isolate, 8/16 (5)
**Temocillin**	93% (BSAC systemic, ≤8 mg/L), 99.7% (BSAC urine, ≤32 mg/L), n=613, Hong Kong, vs.<br>86% piperacillin/tazobactam, 75.4% ceftriaxone, 83.5% ceftazidime, 78.3% gentamicin, 99.2% amikacin, 81.1% cefepime, 88.8% colistin, 89.4% tigecycline, 92.8% fosfomycin, 99.7% meropenem, 99.0% ertapenem (6)<br><br>99.6% (at ≤8 mg/L), 100% at (at ≤32 mg/L), n=762, France (7)<br><br>61.8% (at ≤8 mg/L), 91.0% (at ≤32 mg/L), n=400, Poland, 8/32, vs.	Not active	Not active

	28.5% piperacillin/tazobactam, 26.3% ceftazidime, 43% gentamicin, 57.3% amikacin, 77.0% colistin, 40.5% tigecycline, 40.0% fosfomycin, 88.8% meropenem, 77.0% ertapenem (8).<br><br>91.9% % (at ≤16 mg/L), n=652, Belgian ICU (9)		
**Cefiderocol**	97.2% (EUCAST, ≤ 2 mg/L), n=146, Germany, 0.12/1, vs.<br>100% ceftazidime-avibactam, 100% meropenem, 91.8% ceftolozane-tazobactam, 91.1% amikacin, 89% cefepime, 84.2% ciprofloxacin, 83.6% ceftazidime, 82.2% aztreonam, 75.3% colistin (10).	100%, n=13, Germany, 0.06/0.12, vs.<br>61.5% meropenem, 46.2% amikacin and 38.5% to ciprofloxacin (10).	100%, n=54, Germany, 0.12/0.5, vs.<br>98.1% ceftazidime-avibactam, 98.1% ceftolozane-tazobactam, 98.1% amikacin, 92.6% meropenem, 90.7% cefepime, 88.9% colistin, 87% ceftazidime, 83.3% aztreonam, 79.6% ciprofloxacin (10).
**Ceftazidime/ avibactam**	99% (EUCAST ≤8 mg/L), n=21 850, 18 European countries, 0.12/0.5, vs.<br>74.6% ceftazidime, 79.5% piperacillin/ tazobactam, 74.9% aztreonam, 96.3% meropenem, 83.1% colistin, 71.9% levofloxacin (11).<br><br>*K. pneumoniae*<br>99.9% (EUCAST, ≤8 mg/L), n=6041, USA, 0.12/0.25 (12)	35% (at ≤8 mg/L), n=443 (pneumonia patients), USA, 16/>32vs.<br>37.5% ceftazidime, 32.3% piperacillin/ tazobactam, 41.5% meropenem, 45.6% gentamicin, 36.1% levofloxacin, 93.7% colistin (13).	97.1% (EUCAST ≤8 mg/L), n=6210, USA, 2/4 (12), vs.<br>97.0% ceftolozane/ tazobactam, 83.5% ceftazidime, 79.1% piperacillin/ tazobactam, 78.2% meropenem, 99.7% colistin, 63.5% levofloxacin (12).
**Ceftolozane/ tazobactam**	94.5% (EUCAST, ≤2 mg/L, n=24266, Western Europe, 0.25/1, vs.<br>81.1% ceftazidime, 76.9% levofloxacin, 81.7% colistin, 98.3% meropenem (14).<br><br>79.4% (EUCAST, ≤2 mg/L, n=6316, Eastern Europe, 0.25/32.<br>vs.<br>57.0% ceftazidime, 56.3% levofloxacin, 84.1% colistin, 91.7% meropenem (14).	13.5% (at ≤2 mg/L), n=318, Asia Pacific vs.<br>27.9% amikacin, 99.2% colistin, 14.4% cefepime, 17.5% levofloxacin, 15.1% meropenem, 12.6% piperacillin/ tazobactam (16)	97.0% (EUCAST susceptible breakpoint ≤4 mg/L, n=6210, USA, 0.5/2, vs.<br>97.1% ceftazidime/ avibactam (12).<br><br>94.1% (EUCAST ≤2 mg/L), n=5084, Western Europe, 0.5/2,vs. 79.7% ceftazidime, 76.7% piperacillin/tazobactam, 67.4% levofloxacin, 79.0% meropenem, 99.4% colistin, tobramycin 88.4% (14).

	E. coli<br>94.7% (EUCAST, ≤2 mg/L, n=494, Brazil, 0.25/0.5, vs.<br>78.1% ceftazidime, 71.7% ceftriaxone, 55.1% ciprofloxacin, 99.4% colistin, 99.0% meropenem<br>(15).<br><br>*K. pneumoniae*<br>95.3% (EUCAST susceptible breakpoint ≤2 mg/L, n=6041, USA, 0.25/1 (12).		80.9% (EUCAST, ≤2 mg/L, n=2419, Eastern Europe, 1/>32, vs. 62.8.% ceftazidime, 57.4% piperacillin/tazobactam, 47.7% levofloxacin, 54.5% meropenem, 99.1% colistin, tobramycin 70.9% (14).<br><br>97.0 % (CLSI, ≤4/4 mg/L, n=3851, USA 0.5/2, vs.<br>85.1% ceftazidime, 80.4% piperacillin/ tazobactam, 76.6% levofloxacin, 99.2% colistin, 81.8% meropenem (17)<br><br>92.3% (at ≤4 mg/L), n=765. Asia Pacific vs. 94.9% amikacin, 99.6% colistin, 80.4% cefepime, 76.2% levofloxacin, 77.9% meropenem, 74.1% piperacillin/ tazobactam (16)<br><br>90.9% (EUCAST, ≤4 mg/L, n=265, Brazil, 1/4, vs.<br>66.8% ceftazidime, 59.6% piperacillin/ tazobactam, 69.8% ciprofloxacin, 98.9% colistin, 66.0% meropenem (15).
**Imipenem/ relebactam**	95.4%, n=10516, USA, vs.<br>92.1% imipenem, 98.6% meropenem, 94.0% ceftolozane/ tazobactam, 89.7% cefepime, 90.6% piperaclin/ tazobactam, 73.9% ciprofloxacin (18).<br><br>98.4%, n=10465 (19)	45.8% (EUCAST, ≤2 mg/L), n=72, USA, 4/>32, vs.<br>45.8% imipenem, 66.7% amikacin, 40.3% ceftazidime, 31.9% piperacillin/ tazobactam<br>(20)<br><br>51% (CLSI imipenem breakpoint), n=158, USA, 2-4 / >16-4, vs.<br>49% imipenem (21)	93.9% (CLSI imipenem breakpoint), n=2732, USA, vs.<br>72.0% imipenem, 77.0% meropenem, 94.7% ceftolozane/ tazobactam, 76.9% ceftazidime, 75.6% cefepime, 70.2% piperacilin/ tazobactam, 65.7% ciprofloxacin, 63.1% aztreonam, 96% amikacin, 99.6% colistin (18).<br><br>98% (CLSI imipenem breakpoint), n=490, USA, 0.5-4/ 2-4, vs.<br>70% imipenem (21)

			97.3% (EUCAST imipenem breakpoint), n= 1445, Spain, 0.5/1, vs. 73.5% piperacillin/ tazobactam, 79.7%ceftazidime, 79.4% cefepime, 94.2% ceftazidime/avibactam, 61.6% ciprofloxacin, 91.6% amikacin (22)<br><br>94.4% (breakpoint?), n=1245, 22 European countries (19)

Table S2. Proportion (%) susceptible in various antimicrobial resistant isolates.

	% susceptible (breakpoint used), number of isolates tested, MIC 50/90 value (when available) in mg/L, vs. % susceptible of other antibiotics from the same study when available (reference)		
	**Extended spectrum beta-lactamases**	**Carbapenem non-susceptible**	**Others**
**Plazomicin**	*E. coli*<br>100% (FDA breakpoint, ≤2 mg/L), n=539, n.a. (3)	*Enterobacterales*<br>95.8% (FDA breakpoint, ≤2 mg/L), n=98, n.a., vs.<br>78.6% amikacin, 45.9% gentamicin, 24,2% tobramycin (1)<br><br>85% (FDA breakpoint, ≤2 mg/L), n=227, 0.25/128 (2)<br><br>KPC 92.9% (FDA breakpoint, ≤2 mg/L), , n=113, ≤0.25/2 (2)<br>MBL 40.5% (FDA breakpoint, ≤2 mg/L), n=37, 128/128 (2)<br>OXA-48 87% (FDA breakpoint, ≤2 mg/L), n=54, 0.25/16 (2)<br><br>*K. pneumoniae*<br>97.6% (FDA breakpoint, ≤2 mg/L), n=697, 0.25/1 (23)	Aminoglycosides modifying enzymes genes present<br>99% (FDA breakpoint, ≤2 mg/L), n=728, 0.25/1 (2)<br><br>Non-susceptible to ≥ 2 aminoglycosides<br>96.8% (FDA breakpoint, ≤2 mg/L), n=680 (1)<br><br>Non-susceptible to 3 aminoglycosides<br>52.2% (FDA breakpoint, ≤2 mg/L), n=23 (1)<br><br>*Enterobacterales* non-susceptible to all but 2 antimicrobial classes<br>89.2% (FDA breakpoint, ≤2 mg/L), n=102 (1)

			Colistin-resistant *Enterobacterales* isolates<br>89.5% (FDA breakpoint, ≤2 mg/L), n=95<br>Isolates harboring *mcr-1* 100% (FDA breakpoint, ≤2 mg/L), n=21, n.a. (24)
**Eravacycline**	*E. coli*<br>% n.a., n= 141, 0.25/0.5 (4)	*E. coli*<br>98% (CLSI, ≤0.5 mg/L), n=343, 0.125/0.5, vs. 78% amikacin, 18% levofloxacin, 41% meropenem, 71% imipenem (25)<br>KPC 98% (CLSI, ≤0.5 mg/L), n =55 (25)<br>MBL 95% (CLSI, ≤0.5 mg/L), n=64 (25)<br>OXA-48 100% (CLSI, ≤0.5 mg/L), n=46 (25)	Colistin-resistant<br>*E. coli mcr-1* positive<br>% na, n=16, 0.25/0.5 (26)<br>vs.<br>Tetracycline >32/>32 (intrinsic), tigecycline 0.25/0.5
**Temocillin**	*E. coli, Klebsiella spp., and Proteus* spp.<br>83.9% (BSAC systemic, ≤8 mg/L), n=118, Hong Kong, 8/16 (6)<br><br>*Enterobacterales*<br>66.2% (resistant if MIC≤ 32 µg/ml.), n =77, Belgium, vs.<br>98.7% meropenem (9)	*K. pneumonia KPC* producing<br>8.6% (BSAC systemic, ≤8 mg/L), 84.6% (BSAC urine, ≤32 mg/L), n=280, 24/48 (27)<br><br>*K. pneumoniae* and *E.coli KPC* producing<br>91% (BSAC urine, ≤32 mg/L), n=33, 32/32 (28)<br><br>*E. coli*, *K. pneumoniae* and *Enterobacter* spp.<br>KPC 50.8% (at ≤8 mg/L), 93.9% (at ≤16 mg/L), n =669<br>VIM 0% (at ≤8 mg/L, 2.2% (at ≤16 mg/L), n=138<br>IMP 0% (at ≤8 mg/L), 100% (at ≤16 mg/L), n=1<br>OXA-48 0% (at ≤8 mg/L), 3.7% (at ≤16 mg/L), n=108 (typical) (29)<br><br>*E.coli*, *K. pneumoniae* and *Enterobacter* spp.<br>Carbapenem non-susceptible without carbapenemases genes (BSAC systemic, ≤8 mg/L), 15.6%, n=77, vs.<br>100% ceftazidim/ avibactam (30)	

**Cefiderocol**	*Enterobacterales* (TEM, ESBL, SHV ESBL, CTX-M, PER or AmpC)<br>2/ 8, n= 43 (31)	*Enterobacterales*<br>83.3% (EUCAST, ≤ 2 mg/L), n= 30, 1/4 (10)	
		A. baumannii, 100%, n=7, (10) 0.25/0.25, vs. 0% ciprofloxacin, 100% colistin, 85.7% amikacin.	
		P. aeruginosa 100% (≤ 2 mg/L), n = 27, 0.5/2, vs.<br>0% ceftazidime, 7% cefepime, 0% ciprofloxacin, 8% ceftazidime-avibactam, 26% ceftolozane/ tazobactam, 22% aztreonam, 40.9% amikacin, 96% colistin (31)	
**Ceftazidime/ avibactam**	*K. pneumoniae*<br>100% (EUCAST, ≤8 mg/L), n=614<br>USA, 0.25/0.5 (12)	*E. coli*<br>82% (CLSI, ≤8/4 mg/L), n=343, 0.5/>256, vs. 78% amikacin, 18% levofloxacin, 41% meropenem, 71% imipenem (25)<br>KPC 98% (CLSI, ≤8/4 mg/L), n =55 (25)<br>MBL 8% (CLSI, ≤8/4 mg/L), n=64 (25)<br>OXA-48 91% ((CLSI, ≤8/4 mg/L), n=46 (25)<br><br>*E.coli*, *K. pneumonia* and *Enterobacter* spp.<br>Without carbapenemases genes<br>100%, n=77 (30)<br><br>*K. pneumonia*<br>99.2% (EUCAST, ≤8 mg/L), n=161, USA, 1/2, vs.<br>0% ceftolozane–tazobactam, 8.7% levofloxacin, 44.7%, gentamicin, 42.9% 82.4% colistin (12).<br><br>*K. pneumonia*<br>MBL 2%, n=200, 256/>256,<br>vs.	Susceptible to ≤2 classes only:<br>*P.aeruginosa*<br>80.1% (EUCAST, ≤8 mg/L), n=810<br>USA, 4/16 (12)<br><br>Colistin resistant:<br>*Escherichia coli mcr-1*<br>100% (CLSI breakpoint, ≤2/4 mg/L), n=17, China<br>vs.<br>100% meropenem, 100% tigecycline (34)

		88% colistin, 11% aztreonam. 14% levofloxacin (32).<br><br>*Enterobacterales* non CPE producers<br>96% (CLSI, ≤8/4 mg/L), n=151,<br>*Enterobacterales* CPE producers<br>47.1% (CLSI, ≤8/4 mg/L), n=1041, vs.<br>7.2% ciprofloxacin, 27.3% gentamicin, 38.5% amikacin, 59.3% tigecycline, 79.8% colistin (33)	
		A. baumannii<br>2.5% (CLSI, ≤8 mg/L), n=40, vs.<br>72.5% colistin, 10% tobramycin (36).	
		P. aeruginosa<br>83.3% (EUCAST, ≤4 mg/L), n=102, vs.<br>53.0% piperacillin/ tazobactam, 68.6% ceftazidime, 48.0% cefepime, 83.3% ceftolozane/ tazobactam (35).	
**Ceftolozane/ tazobactam**	*K. pneumonia*<br>82.9% (EUCAST, ≤2 mg/L), n=614, 1/8 (12)<br><br>*Enterobacterales*<br>82.4% % (CLSI, ≤2 mg/L), n=3599, 0.5/16, vs.<br>7.8% cefepime, 98.7% meropenem, 95.4% amikacin (37)<br><br>98% (CLSI, ≤2 mg/L), n.a./1 n=116 (38)	*K. pneumonia*<br>0% (EUCAST, ≤2 mg/L), n=161 USA, >16/>16 (12)<br><br>*Enterobacterales*<br>2.6% (CLSI, ≤4 mg/L), n=1018, >64/>64 vs. 77.5% ceftazidime/ avibactam, vs. 78.1% colistin n=1018 (39)	MDR (resistant to ≤3 antibiotic classes)<br>*K. pneumoniae*<br>54% (CLSI, ≤2/4 mg/L), n=26, Lebanon (38)<br>*P. aeruginosa*<br>43% (CLSI, ≤4/4 mg/L), n=69, Lebanon (38)<br><br>Non-MBL MDR<br>64.4% (EUCAST susceptible breakpoint ≤4 mg/L, n=160, 2/64.<br>Vs. 80.0% ceftazidim/ avibactam, 96.3% colistin (40)
		Acinetobacter spp.<br>0.4% (at ≤2 mg/L), n=269, vs.<br>15.7% amikacin, 99.2% colistin, 14.4% cefepime, 4% levofloxacin, 12.6% piperacillin/ tazobactam (16)	
		P. aeruginosa<br>87.6 % (CLSI, ≤4 mg/L), n=699, 1/8, vs.<br>90.3% amikacin, 98.7% colistin (17)	XDR (susceptible to ≤2 classes only)<br>*P. aeruginosa*<br>80.7% (EUCAST, ≤4 mg/L), n=810 USA, 2/16

	92.5 % (CLSI, ≤4 mg/L), n=466, 1/4, vs. 80.7% amikacin, 94.0% colistin (42)<br><br>72.8% (at ≤4 mg/L), n=180, vs. 79.9% amikacin, 99.4% colistin, 48.3% cefepime, 37.6% ciprofloxacin, 43.1% piperacillin/ tazobactam (16)<br><br>82.4% (EUCAST, ≤4 mg/L), n=102, vs. 53.0% piperacillin/ tazobactam, 68.6% ceftazidime, 48.0% cefepime,83.3% ceftazidime/ avibactam (35)	vs. 80.1% ceftazidime/ tazobactam (12)<br><br>Colistin resistant *P. aeruginosa* 88.9% (CLSI breakpoint, ≤2/4 mg/L), n-9, 1/4 (41)
**Meropenem/ vaborbactam**	*Enterobacterales*<br>98 % (EUCAST, ≤8 mg/L), n=152, 0.06/2 (43)<br>KPC 100% (EUCAST, ≤8 mg/L), 0.03/0.5, n=124 (43)<br>MBL and OXA-48 57.1% (EUCAST, ≤8 mg/L), 8/8 n=7 (43)<br>CPE (EUCAST, ≤8 mg/L), 97.8%, 0.03/1, n=134 (43)	
**Imipenem/ relebactam**	*Enterobacterales* imipenem non-susceptible 66.7% (CLSI breakpoint for imipenem ≤1 mg/L),n=72, 1/2 (44).<br><br>*K. pneumoniae*<br>n.a. (no breakpoint), n=200, ≤0.25/0.5, (45)<br><br>*K. pneumoniae* KPC (imipenem non-susceptible)<br>98.6% (CLSI breakpoint for imipenem ≤1 mg/L), n=138 (19)<br>97% (CLSI imipenem breakpoint), n=111, USA, 0.25-4 /1-4 , vs. 9% imipenem (21)	Not susceptible to all β-Lactam and to fluoroquinolones<br>*Enterobacterales*<br>82.4% (FDA breakpoint ≤1 mg/n=102, vs. 0% imipenem, 0% meropenem, 79.4% amikacin, 84.3% colistin<br>*P. aeruginosa*<br>62.2%, n=230 vs. 0% imipenem, 0% meropenem, 67.5% ceftolozane/ tazobactam, 84.8%% amikacin, 98.7% colistin (18)

	OXA-48 15% (CLSI breakpoint for imipenem ≤1 mg/L), n=20, 4-4/32-4 vs. 90% ceftazidime/ avibactam (46)	
	P. aeruginosa imipenem non-susceptible 74.4% (breakpoint for imipenem ≤1 mg/L), n=469, (72% carried MBL and non-susceptible to imipenem-relebactam) (19).<br><br>80.5%% (CLSI imipenem breakpoint, ≤1 mg/L), n=251, USA, 2/4 (20).<br><br>78.5% (CLSI breakpoint for imipenem ≤1 mg/L), n=191, 2/4 (44).	

References

1. Castanheira M, Sader HS, Mendes RE, Jones RN. Activity of plazomicin tested against enterobacterales isolates collected from U.S. hospitals in 2016-2017: Effect of different breakpoint criteria on susceptibility rates among aminoglycosides. Antimicrob Agents Chemother. 2020;64(5).
2. Castanheira M, Deshpande LM, Woosley LN, Serio AW, Krause KM, Flamm RK. Activity of plazomicin compared with other aminoglycosides against isolates from European and adjacent countries, including Enterobacteriaceae molecularly characterized for aminoglycoside-modifying enzymes and other resistance mechanisms. J Antimicrob Chemother. 2018;73(12):3346–54.
3. Zhanel GG, Adam HJ, Baxter MR, Fuller J, Nichol KA, Denisuik AJ, et al. 42936 pathogens from Canadian hospitals: 10 years of results (2007-16) from the CANWARD surveillance study. J Antimicrob Chemother. 2019;74:iv5–21.
4. Zhanel GG, Baxter MR, Adam HJ, Sutcliffe J, Karlowsky JA. In vitro activity of eravacycline against 2213 Gram-negative and 2424 Gram-positive bacterial pathogens isolated in Canadian hospital laboratories: CANWARD surveillance study 2014–2015. Diagn Microbiol Infect Dis. 2018;91(1):55–62.
5. Morrissey I, Olesky M, Hawser S, Lob SH, Karlowsky JA, Corey GR, et al. In vitro activity of eravacycline against Gram-negative bacilli isolated in clinical laboratories worldwide from 2013 to 2017. Vol. 64, Antimicrobial Agents and Chemotherapy. 2020.
6. Ip M, Lai CK, Fung KSC, Wong KT, Zhu C, Van de Velde S, et al. Activity of temocillin and 15 other agents, including fosfomycin and colistin, against Enterobacteriaceae in Hong Kong. Eur J Clin Microbiol Infect Dis. 2017;36(12):2491–4.
7. Alexandre K, Réveillon-Istin M, Fabre R, Delbos V, Etienne M, Pestel-Caron M, et al. Temocillin against Enterobacteriaceae isolates from communityacquired urinary tract infections: Low rate of resistance and good accuracy of routine susceptibility testing methods. J Antimicrob Chemother. 2018;73(7):1848–53.
8. Kuch A, Zieniuk B, Żabicka D, Van de Velde S, Literacka E, Skoczyńska A, et al. Activity of temocillin against ESBL-, AmpC-, and/or KPC-producing Enterobacterales isolated in Poland. Eur J Clin Microbiol Infect Dis. 2020;39(6):1185–91.
9. Glupczynski Y, Huang TD, Berhin C, Claeys G, Delmée M, Ide L, et al. In vitro activity of temocillin against prevalent extended-spectrum beta-lactamases producing Enterobacteriaceae from Belgian intensive care units. Eur J Clin Microbiol Infect Dis. 2007;
10. Kresken M, Korte-Berwanger M, Gatermann SG, Pfeifer Y, Pfennigwerth N, Seifert H, et al. In vitro activity of cefiderocol against aerobic Gram-negative bacterial pathogens from Germany. Int J Antimicrob Agents. 2020;56(4).
11. Stone GG, Seifert H, Nord CE. In vitro activity of ceftazidime-avibactam against Gram-negative isolates collected in 18 European countries, 2015–2017. Int J Antimicrob Agents. 2020;56(3).
12. Sader HS, Carvalhaes CG, Streit JM, Doyle TB, Castanheira M. Antimicrobial Activity of Ceftazidime-Avibactam, Ceftolozane-Tazobactam and Comparators Tested Against Pseudomonas aeruginosa and Klebsiella pneumoniae Isolates from United States Medical Centers in 2016–2018 . Microb Drug Resist. 2020;
13. Sader HS, Castanheira M, Flamm RK. Antimicrobial activity of ceftazidime-avibactam against Gram-negative bacteria isolated from patients hospitalized with pneumonia in U.S. medical centers, 2011 to 2015. Antimicrob Agents Chemother. 2017;61(4).
14. Sader HS, Carvalhaes CG, Duncan LR, Flamm RK, Shortridge D. Susceptibility trends of ceftolozane/tazobactam and comparators when tested against European Gram-negative bacterial surveillance isolates collected during 2012-18. J Antimicrob Chemother. 2020;75(10):2907–13.
15. Beirão EM, Rodrigues S da S, Andrade TK de, Serra FB, Paula MDN de, Polis TJB, et al. Activity of ceftolozane-tazobactam and comparators against gram-negative bacilli: Results from the study for monitoring antimicrobial resistance trends (SMART – Brazil; 2016–2017). Brazilian J Infect Dis.

2020;24(4):310–21.
16. Kuo SC, Liu CE, Lu PL, Chen YS, Lu MC, Ko WC, et al. Activity of ceftolozane-tazobactam against Gram-negative pathogens isolated from lower respiratory tract infections in the Asia-Pacific region: SMART 2015-2016. Int J Antimicrob Agents. 2020;
17. Shortridge D, Castanheira M, Pfaller MA, Flamm RK. Ceftolozane-tazobactam activity against Pseudomonas aeruginosa clinical isolates from U.S. hospitals: Report from the PACTS antimicrobial surveillance program, 2012 to 2015. Antimicrob Agents Chemother. 2017;61(7).
18. Karlowsky JA, Lob SH, Raddatz J, DePestel DD, Young K, Motyl MR, et al. In Vitro Activity of Imipenem/Relebactam and Ceftolozane/Tazobactam Against Clinical Isolates of Gram-negative Bacilli With Difficult-to-Treat Resistance and Multidrug-resistant Phenotypes—Study for Monitoring Antimicrobial Resistance Trends, United States 2015–2017. Clin Infect Dis. 2020;
19. Lob SH, Karlowsky JA, Young K, Motyl MR, Hawser S, Kothari ND, et al. In vitro activity of imipenem-relebactam against resistant phenotypes of Enterobacteriaceae and Pseudomonas aeruginosa isolated from intraabdominal and urinary tract infection samples - SMART Surveillance Europe 2015-2017. Vol. 69, Journal of Medical Microbiology. 2020. p. 207–17.
20. Lob SH, Hackel MA, Kazmierczak KM, Young K, Motyl MR, Karlowsky JA, et al. Vitro activity of imipenem-relebactam against gram-negative eskape pathogens isolated by clinical laboratories in the United States in 2015 (results from the smart global surveillance program). Antimicrob Agents Chemother. 2017;61(6).
21. Lapuebla A, Abdallah M, Olafisoye O, Cortes C, Urban C, Landman D, et al. Activity of imipenem with relebactam against gram-negative pathogens from New York City. Antimicrob Agents Chemother. 2015;59(8):5029–31.
22. Fraile-Ribot PA, Zamorano L, Orellana R, Del Barrio-Tofiño E, Sánchez-Diener I, Cortes-Lara S, et al. Activity of imipenem-relebactam against a large collection of pseudomonas aeruginosa clinical isolates and isogenic β-lactam-resistant mutants. Antimicrob Agents Chemother. 2020;64(2).
23. Jacobs MR, Good CE, Hujer AM, Abdelhamed AM, Rhoads DD, Hujer KM, et al. ARGONAUT II study of the in vitro activity of plazomicin against carbapenemase-producing klebsiella pneumoniae. Antimicrob Agents Chemother. 2020;64(5).
24. Denervaud-Tendon V, Poirel L, Connolly LE, Krause KM, Nordmann P. Plazomicin activity against polymyxin-resistant Enterobacteriaceae, including MCR-1-producing isolates. J Antimicrob Chemother. 2017;72(10):2787–91.
25. Johnston BD, Thuras P, Porter SB, Anacker M, VonBank B, Vagnone PS, et al. Activity of cefiderocol, ceftazidime-avibactam, and eravacycline against carbapenem-resistant escherichia coli isolates from the united states and international sites in relation to clonal background, resistance genes, coresistance, and region. Antimicrob Agents Chemother. 2020;64(10).
26. Fyfe C, LeBlanc G, Close B, Nordmann P, Dumas J, Grossman TH. Eravacycline is active against bacterial isolates expressing the polymyxin resistance gene mcr-1. Vol. 60, Antimicrobial Agents and Chemotherapy. 2016. p. 6989–90.
27. Tsakris A, Koumaki V, Politi L, Balakrishnan I. Activity of temocillin against KPC-producing Enterobacteriaceae clinical isolates. Int J Antimicrob Agents. 2020;55(1).
28. Adams-Haduch JM, Potoski BA, Sidjabat HE, Paterson DL, Doi Y. Activity of temocillin against KPC-producing Klebsiella pneumoniae and Escherichia coli. Vol. 53, Antimicrobial Agents and Chemotherapy. 2009. p. 2700–1.
29. Woodford N, Pike R, Meunier D, Loy R, Hill R, Hopkins KL. In vitro activity of temocillin against multidrug-resistant clinical isolates of Escherichia coli, Klebsiella spp. and Enterobacter spp., and evaluation of high-level temocillin resistance as a diagnostic marker for OXA-48 carbapenemase. Vol. 69, Journal of Antimicrobial Chemotherapy. 2014. p. 564–7.
30. Mutters NT, Zimmermann S, Kaase M, Mischnik A. Activity of temocillin, mecillinam, ceftazidime,

and ceftazidime/avibactam against carbapenem-non-susceptible Enterobacteriaceae without carbapenemase production. Eur J Clin Microbiol Infect Dis. 2015;34(12):2429–37.

31. Jacobs MR, Abdelhamed AM, Good CE, Rhoads DD, Hujer KM, Hujer AM, et al. ARGONAUT-I: Activity of cefiderocol (s-649266), a siderophore cephalosporin, against gram-negative bacteria, including carbapenem-resistant nonfermenters and enterobacteriaceae with defined extended-spectrum β-lactamases and carbapenemases. Antimicrob Agents Chemother. 2019;63(1).
32. Rossolini GM, Stone GG. Assessment of the in vitro activity of ceftazidime/avibactam against a global collection of multidrug-resistant Klebsiella spp. from the INFORM surveillance programme (2015–2017). Int J Antimicrob Agents. 2020;56(3).
33. Sonnevend Á, Ghazawi A, Darwish D, Barathan G, Hashmey R, Ashraf T, et al. In vitro efficacy of ceftazidime-avibactam, aztreonam-avibactam and other rescue antibiotics against carbapenem-resistant Enterobacterales from the Arabian Peninsula. Int J Infect Dis. 2020;
34. Jiang B, Du P, Jia P, Liu E, Kudinha T, Zhang H, et al. Antimicrobial Susceptibility and Virulence of mcr-1-Positive Enterobacteriaceae in China, a Multicenter Longitudinal Epidemiological Study. Front Microbiol. 2020;11.
35. Mirza H, Elvan H, Koçak, AA, Demirkaya H, Yayla B, Güçlü AÜ, et al. In vitro activity of ceftolozane-tazobactam and ceftazidime-avibactam against clinical isolates of meropenem-non-susceptible Pseudomonas aeruginosa: A two-centre study. J Glob Antimicrob Resist. 2020;334–8.
36. Mataracı Kara E, Yılmaz M, Özbek Çelik B. In vitro activities of ceftazidime/avibactam alone or in combination with antibiotics against multidrug-resistant Acinetobacter baumannii isolates. J Glob Antimicrob Resist. 2019;17:137–41.
37. Karlowsky JA, Kazmierczak KM, Young K, Motyl MR, Sahm DF. In vitro activity of ceftolozane/tazobactam against phenotypically defined extended-spectrum β-lactamase (ESBL)-positive isolates of Escherichia coli and Klebsiella pneumoniae isolated from hospitalized patients (SMART 2016). Diagn Microbiol Infect Dis. 2020;96(4).
38. Araj GF, Berjawi DM, Musharrafieh U, Beayni NKE. Activity of ceftolozane/tazobactam against commonly encountered antimicrobial resistant Gram-negative bacteria in Lebanon. J Infect Dev Ctries. 2020;14(6):559–64.
39. Karlowsky JA, Hackel MA, Bouchillon SK, Sahm DF. In Vitro Activity of WCK 5222 (Cefepime-Zidebactam) against Worldwide Collected Gram-Negative Bacilli Not Susceptible to Carbapenems. Antimicrob Agents Chemother. 2020;64(12).
40. Galani I, Papoutsaki V, Karantani I, Karaiskos I, Galani L, Adamou P, et al. In vitro activity of ceftolozane/tazobactam alone and in combination with amikacin against MDR/XDR Pseudomonas aeruginosa isolates from Greece. J Antimicrob Chemother. 2020;75(8):2164–72.
41. Shortridge D, Pfaller MA, Arends SJR, Raddatz J, Depestel DD, Flamm RK. Comparison of the in Vitro Susceptibility of Ceftolozane-Tazobactam with the Cumulative Susceptibility Rates of Standard Antibiotic Combinations When Tested against Pseudomonas aeruginosa from ICU Patients with Bloodstream Infections or Pneumonia. Open Forum Infect Dis. 2019;6(6).
42. McCracken MG, Adam HJ, Blondeau JM, Walkty AJ, Karlowsky JA, Hoban DJ, et al. Characterization of carbapenem-resistant and XDR Pseudomonas aeruginosa in Canada: results of the CANWARD 2007-16 study. J Antimicrob Chemother. 2019;74:iv32–8.
43. Castanheira M, Doyle TB, Kantro V, Mendes RE, Shortridge D. Meropenem-vaborbactam activity against carbapenem-resistant enterobacterales isolates collected in U.S. Hospitals during 2016 to 2018. Antimicrob Agents Chemother. 2020;64(2).
44. Lob SH, Hackel MA, Kazmierczak KM, Hoban DJ, Young K, Motyl MR, et al. In vitro activity of imipenem-relebactam against gram-negative bacilli isolated from patients with lower respiratory tract infections in the United States in 2015 – Results from the SMART global surveillance program. Diagn Microbiol Infect Dis. 2017;88(2):171–6.

45. Carpenter J, Neidig N, Campbell A, Thornsberry T, Truex T, Fortney T, et al. Activity of imipenem/relebactam against carbapenemase-producing Enterobacteriaceae with high colistin resistance. J Antimicrob Chemother. 2019;74(11):3260–3.
46. Canver MC, Satlin MJ, Westblade LF, Kreiswirth BN, Chen L, Robertson A, et al. Activity of imipenem-relebactam and comparator agents against genetically characterized isolates of carbapenem-resistant Enterobacteriaceae. Antimicrob Agents Chemother. 2019;63(9).
